# Heart rate dynamics during cardio-pulmonary exercise testing are associated with glycemic control in individuals with type 1 diabetes

**DOI:** 10.1371/journal.pone.0194750

**Published:** 2018-04-02

**Authors:** Othmar Moser, Max L. Eckstein, Olivia McCarthy, Rachel Deere, Stephen C. Bain, Hanne L. Haahr, Eric Zijlstra, Tim Heise, Richard M. Bracken

**Affiliations:** 1 Diabetes Research Group, Medical School, Swansea University, Swansea, United Kingdom; 2 Applied Sport, Technology, Exercise and Medicine Research Centre (A-STEM), College of Engineering, Swansea University, Swansea, United Kingdom; 3 Novo Nordisk A/S, Søborg, Denmark; 4 Profil, Neuss, Germany; University of Colorado Denver School of Medicine, UNITED STATES

## Abstract

**Introduction:**

This study investigated the degree and direction (k_HR_) of the heart rate to performance curve (HRPC) during cardio-pulmonary exercise (CPX) testing and explored the relationship with diabetes markers, anthropometry and exercise physiological markers in type 1 diabetes (T1DM).

**Material and methods:**

Sixty-four people with T1DM (13 females; age: 34 ± 8 years; HbA_1c_: 7.8 ± 1% (62 ± 13 mmol.mol^-1^) performed a CPX test until maximum exhaustion. k_HR_ was calculated by a second-degree polynomial representation between post-warm up and maximum power output. Adjusted stepwise linear regression analysis was performed to investigate k_HR_ and its associations. Receiver operating characteristic (ROC) curve was performed based on k_HR_ for groups k_HR_ < 0.20 vs. > 0.20 in relation to HbA_1c_.

**Results:**

We found significant relationships between k_HR_ and HbA_1c_ (*β = -0*.*70*, *P < 0*.*0001*), age (*β = -0*.*23*, *P = 0*.*03)* and duration of diabetes (*β = 0*.*20*, *P = 0*.*04)*. Stepwise linear regression resulted in an overall adjusted *R*^*2*^ of 0.57 (*R = 0*.*79*, *P < 0*.*0001*). Our data revealed also significant associations between k_HR_ and percentage of heart rate at heart rate turn point from maximum heart rate (*β = 0*.*43*, *P < 0*.*0001)* and maximum power output relativized to bodyweight (*β = 0*.*44*, *P = 0*.*001)* (overall adjusted *R*^*2*^ of 0.44 (*R = 0*.*53*, *P < 0*.*0001*)). ROC curve analysis based on k_HR_ resulted in a HbA_1c_ threshold of 7.9% (62 mmol.mol^-1^).

**Conclusion:**

Our data demonstrate atypical HRPC during CPX testing that were mainly related to glycemic control in people with T1DM.

## Introduction

**Fig 1:**
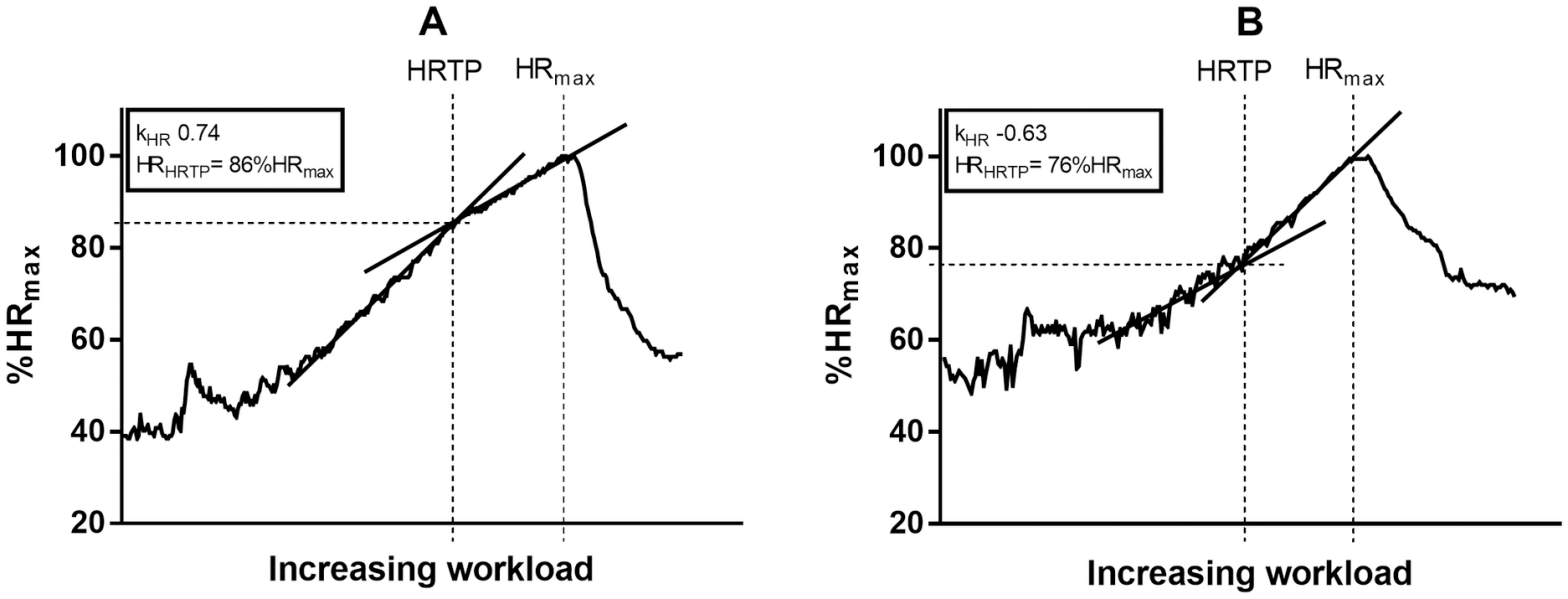
Schematic of the heart rate to performance curve (HRPC) and detection of the heart rate turn point (HRTP) during CPX testing, illustrating a regular HRPC (A) and an inverted HRPC (B). The difference in HRPC translates to a lower heart rate at HRTP (HR_HRTP_) when given as percentage of the maximum heart rate (%HR_max_) (difference 10%). k_HR_ = degree and direction of the heart rate to performance curve. HR_max_ = maximum heart rate.

Cardio-pulmonary exercise (CPX) testing provides detailed diagnostic information about cardio-pulmonary, vascular and musculoskeletal adaptations to physical stressors [1]. Aerobic performance markers, like thresholds (e.g. ventilatory thresholds or the heart rate turn point (HRTP)) are recommended to accurately prescribe individualized exercise intensity [2]. These thresholds relativized to maximum oxygen consumption (VO_2max_) serve as sensitive markers to analyze effects of exercise training in both healthy individuals and patients [3]. As an example the HRTP, which is based on findings from Conconi and colleagues, was significantly associated with the second lactate threshold [4–7]. This heart rate (HR) derived threshold is defined as the intersection of two regression lines of the HR to performance curve (HRPC) between early stages of CPX testing (peri-first lactate turn point (LTP_1_)) and maximum power output (P_max_), determined from a second-degree polynomial representation satisfying the condition of least error squares (Fig 1) [8].

From a physiological point of view the main cause for the HRTP can be seen in the β_1_-receptor sensitivity to the catecholamine response [9]. Hofmann et al. investigated the response to a single dose of the β_1_-selective antagonist bisoprolol in healthy individuals [10]. This study revealed a significant association between the response to the antagonist and different patterns of the HRPC. A regular HRPC translated to inverted HRPC when using the β_1_-antagonist. However, an inverted HRPC under placebo did not change its pattern under a β_1_-antagonist application. This shows that the inverted HRPC in placebo conditions is caused by a reduced β1-receptor sensitivity.

Inter-individual differences in HRPC were observed in healthy individuals and in different groups of patients [8]. In the general population, approximately 86% of people show regular deflections of HRPC across a sub-maximal (HRTP) to maximal (P_max_) continuum; however, 8% reveal inverted deflections and 6% display linear increases in HRPC.

Chronotropic incompetence (CI) is the inability of the HR to increase in proportion to raised metabolic demand, and is found mainly in people with coronary artery disease. CI is a strong and independent predictor of overall mortality [11]. Interestingly, this non-physiological cardiac response was also reported in people with type 2 diabetes, where its origin is not fully understood. Diabetes *per se* and/or disease-related comorbidities as well as physiological anomalies seem to play a role for CI [12].

Poor glycemic control in people with T1DM may be associated with blunted functional capacity compared to healthy individuals, which is mainly assessed by means of VO_2max_ [13–15]. Some studies suggested that poor glycemic control may alter cardio-respiratory and metabolic responses to exercise, which translates to a general lower functional capacity in people with T1DM [13,15]. Furthermore, it has been shown that people with T1DM have a reduced maximum HR (HR_max_) in comparison to their healthy counterparts [13]. Intriguingly, the blunted effect of HR_max_ was shown to be dependent on glycemic control [16].

It is currently not known if the degree and the direction (k_HR_) of the HRPC during CPX testing is related to glycemic control in people with T1DM. Therefore, the aim of this study was to investigate k_HR_ during CPX testing and explore relationships to diabetes markers, anthropometry and exercise physiological markers in a large group of people with T1DM.

## Material and methods

### Participant characteristics

**Fig 2:**
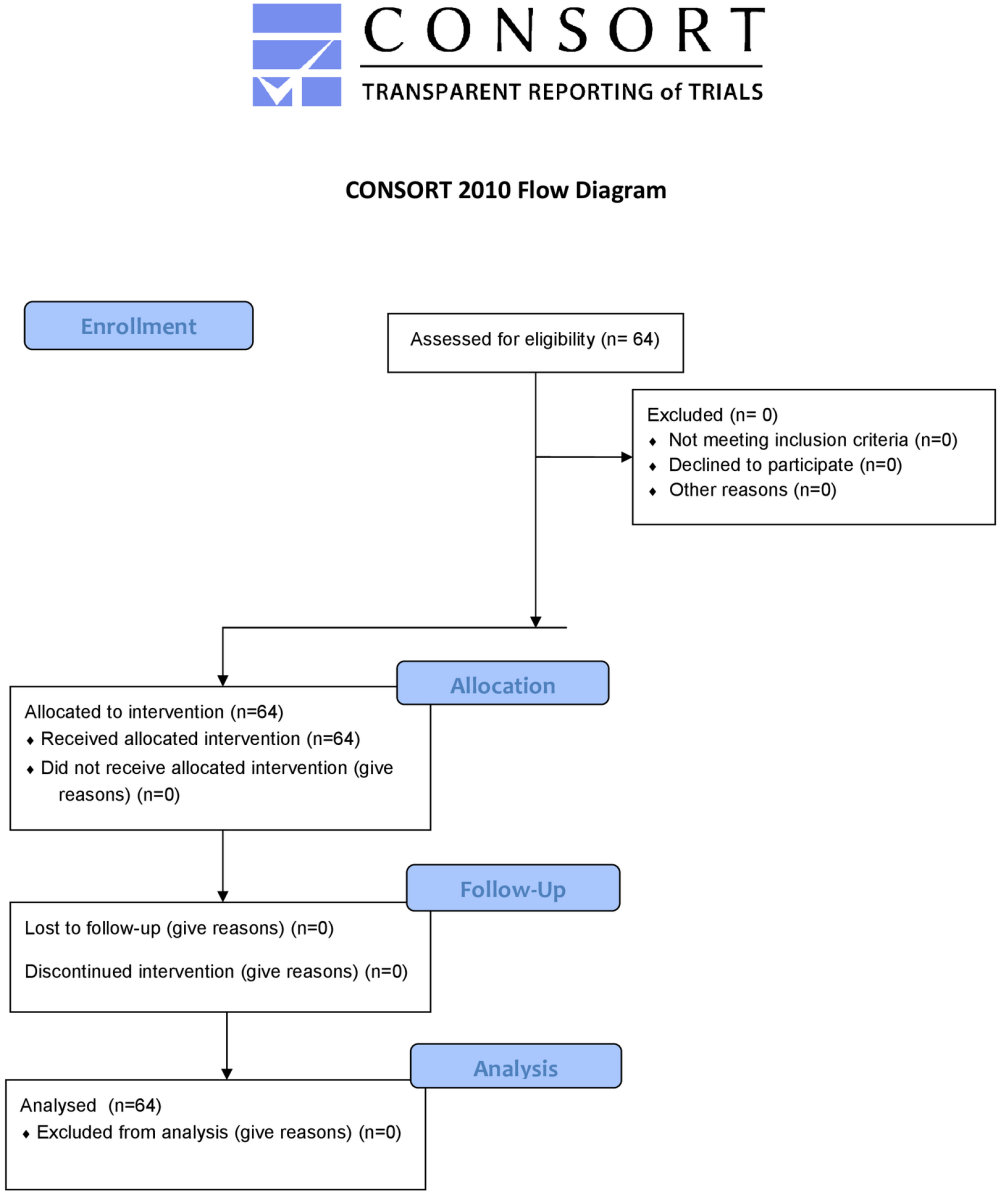
Consort flow diagram.

For this study sixty-four people with T1DM were recruited from October 2012 until March 2013 by advertisement in local newspapers (Table 1, Fig 2):

**Table 1:** Participant characteristics given as mean ± SD and percentage (%).

Characteristic	Total (n = 64)
Age (years)	34 ± 8
Female (n; %)	13 (20)
Male (n; %)	51 (80)
Body mass index (kg/m^2^)	24 ± 2
Duration of diabetes (years)	17 ± 9
HbA_1c_ (% (mmol.mol^-1^))	7.8 ± 1 (62 ± 13)
Total daily dose of insulin (U)	51 ± 15
Multiple daily injections (n; %)	47 (78)
Insulin pump therapy (n; %)	17 (22)
Arterial hypertension	6
Hypothyroidism	5
Hypercholesterolemia	2
Hashimoto thyroiditis	1
ACE inhibitor	6
Levothyroxine	6
Statin	2
Diuretic medication	1
Calcium channel blocker	1
Physical activity (MET min.wk ^1^))	3086 ± 2736
Maximum oxygen uptake (ml.kg^-1^.min^-1^)	37 ± 5

### Consent procedure

Participants gave their written informed consent before any trial related activities. The trial was performed accordingly to the Declaration of Helsinki (DoH) and Good Clinical Practice (GCP) Guidelines. The primary study protocol was approved by the local ethics committee and health authority board. The study protocol was registered with the universal clinical trial registry, number NCT01704417 [17].

### Study procedures

Participants filled in the International Physical Activity Questionnaire (IPAQ) to assess physical activity (MET min/week). Medical history, medications and patients’ characteristics were documented on the day of the CPX testing. Immediately afterwards, HbA_1c_ was measured from a venous blood sample (Automated Glycohemoglobin Analyzer HLC-723G8, Tosoh Europe N.V, Belgium). Venous blood was collected immediately before and after CPX testing to evaluate blood glucose concentration (Super GL Glucose Analyzer, Dr. Müller Gerätebau GmbH, Germany). Participants performed a CPX test until maximum volitional exhaustion on a cycle ergometer (Ergospirometer PowerCube^®^-Ergo, Ganshorn Medizin Electronic, GER) under medical supervision. Participants sat for 3 min (0 watt (W)) on the cycle ergometer before they started the warm-up period of 3 min cycling at an exercise intensity of 30 W for females and 40 W for males. After the warm-up period, the intensity was increased by 30 W for females and 40 W for males every 3 minutes until maximum volitional exhaustion. Finally, an active recovery period was conducted for 1 min.

### Measurements

Pulmonary gas exchange variables were measured continuously. Data were then averaged over 10 seconds to control for artefacts. Blood pressure and HR were measured continuously via an automatic sphygmomanometer and a 12-lead electrocardiogram (Ergospirometer PowerCube^®^-Ergo, Ganshorn Medizin Electronic, GER). The non-invasive anaerobic threshold was defined by the HRTP. HRTP was demarcated as the intersection of two regression lines of HRPC between post-warm-up and P_max_, determined from a second-degree polynomial representation satisfying the condition of least error squares [8]. All measurements were conducted at Profil, Neuss, Germany.

### Statistical analyses

Data were tested for normal distribution via Shapiro-Wilk test. Descriptive statistics included mean and standard deviation for participant’s characteristics. k_HR_ was calculated by a second-degree polynomial representation between post-warm up and P_max_. Stepwise linear regression was used to explore relationships between k_HR_ and diabetes markers (glycemic control (HbA_1c_), total daily dose of insulin (both basal- and bolus insulin), duration of diabetes), anthropometry (height, weight, body mass index (BMI)) and physical activity (IPAQ). Stepwise linear regression was also used between k_HR_ and exercise physiological markers (CPX derived cardio-respiratory markers at HRTP and at P_max_). Stepwise linear regressions were adjusted for gender, BMI, physical activity, total daily dose of insulin, duration of diabetes and blood glucose concentration at the start of CPX testing if not included in the regression model. Logarithmic transformation was performed if data were non-normally distributed. Receiver operating characteristic (ROC) curves based on k_HR_ for groups k_HR_ < 0.20 vs. > 0.20 in relation to HbA_1c_. All statistical analyses were carried out using SPSS V.22.0 statistical software (SPSS, Chicago, Illinois, USA). A sample size of 64 individuals with T1DM resulted in a power (1 –β error probability) of 1.0 for the main outcome analyzed via stepwise linear regression, respectively.

## Results

### Relationships between k_HR_ and HbA_1c_, total daily dose of insulin, duration of diabetes, anthropometry and physical activity

**Fig 3:**
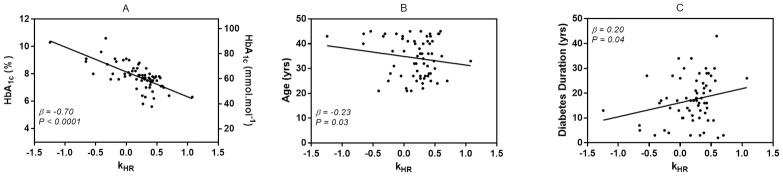
Single plots of the association of k_HR_ and HbA_1c_ (A), age (B) and diabetes duration (C). k_HR_ = degree and direction of the heart rate to performance curve.

We found significant relationships between k_HR_ and HbA_1c_ (*β = -0*.*70*, *P < 0*.*0001*), age (*β = -0*.*23*, *P = 0*.*03) and* duration of diabetes (*β = 0*.*20*, *P = 0*.*04)* (Fig 3). Stepwise linear regression resulted in an overall adjusted *R*^*2*^ of 0.57 (*R = 0*.*79*, *P < 0*.*0001*).

### Relationships between k_HR_ exercise physiological markers

**Fig 4:**
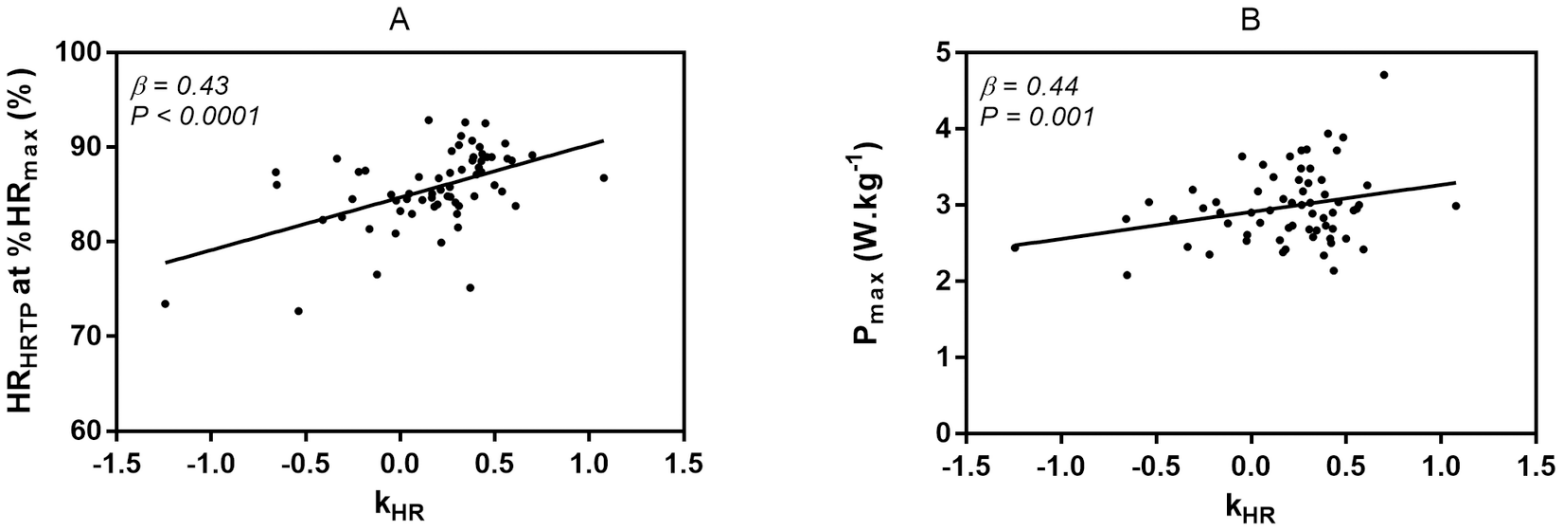
Single plots of the association of k_HR_ and HR_HRTP_ at %HR_max_ (A) and P_max_ (B). k_HR_ = degree and direction of the heart rate to performance curve, HR_HRTP_ at %HR_max_ = heart rate at the heart rate turn point given as percentages of the maximum heart rate, P_max_ = maximum power output relativized to bodyweight.

Our data revealed significant associations between k_HR_ and percentage of HR at HRTP from HR_max_ (*β = 0*.*43*, *P < 0*.*0001)* and P_max_ relativized to bodyweight (*β = 0*.*44*, *P = 0*.*001)*. Stepwise linear regression resulted in an overall adjusted *R*^*2*^ of 0.44 (*R = 0*.*53*, *P < 0*.*0001*) (Fig 4).

### ROC curve analysis based on k_HR_

**Fig 5:**
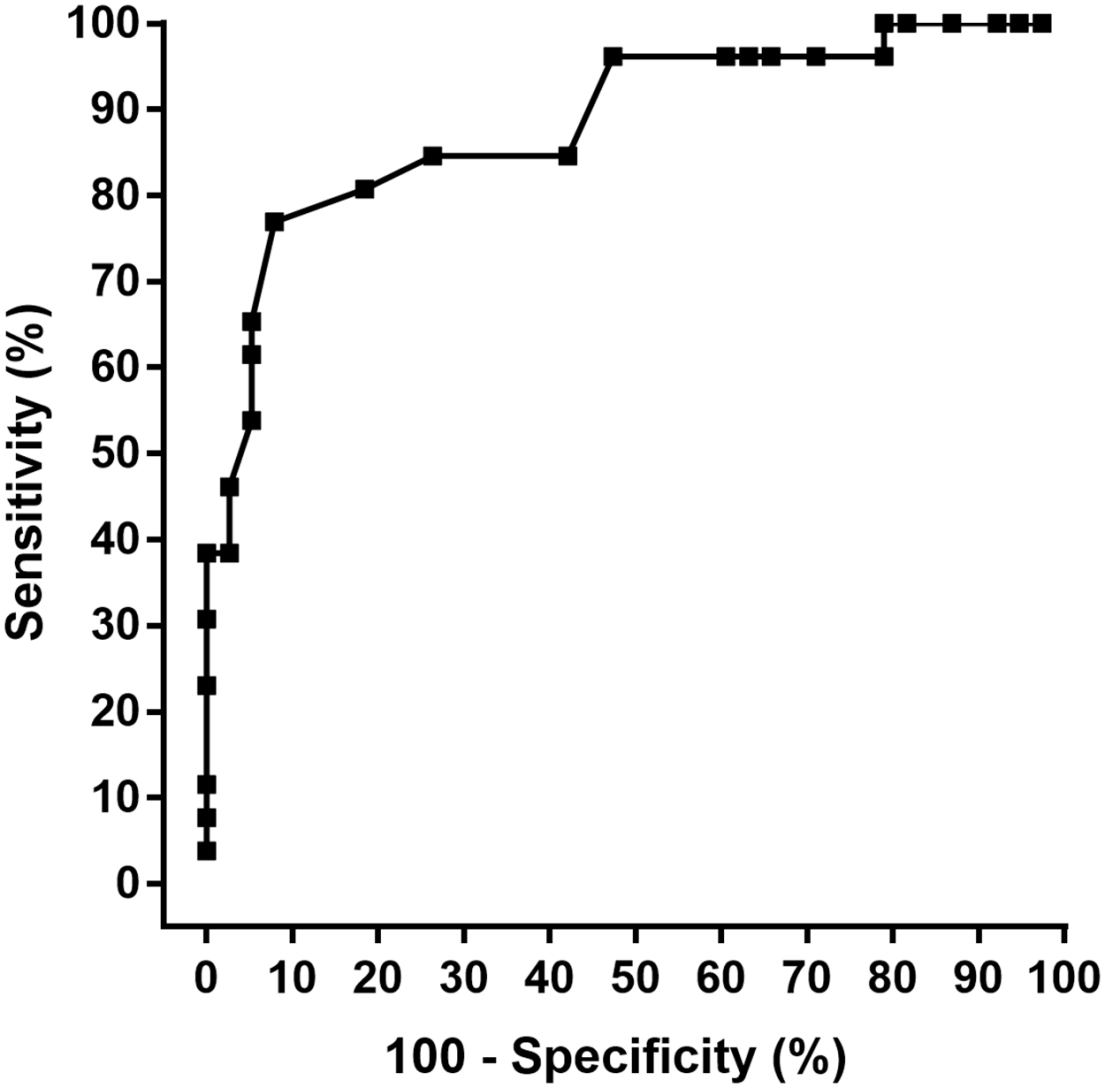
Receiver operating characteristic (ROC) curve analysis of HbA_1c_. The sensitivity is plotted against 100–specificity to indicate accuracy. The optimal value for sensitivity was 81%, which corresponded to a specificity of 82%. This represents a cut off level for HbA_1c_ of 7.9% (63 mmol.mol^-1^).

ROC curve analysis based on k_HR_ for groups k_HR_ < 0.20 vs. > 0.20 resulted in a HbA_1c_ threshold of 7.9% (63 mmol.mol^-1^) (81% sensitivity and 82% specificity) (Fig 5).

## Discussion

This study demonstrated the clear association between poor glycemic control and HR dynamics during CPX testing. Intriguingly, higher HbA_1c_ and its translation to atypical k_HR_ resulted in lower HR responses at the HRTP and lower bodyweight-relativized maximum power output. Several physiological mechanisms might explain these novel findings:

Diabetes specific co-morbidities (e.g. structural myocardial alterations, ventricular and/or arterial stiffness, impaired baroreflex sensitivity and cardiovascular autonomic neuropathy) might minimally contribute to these alterations in k_HR_, as the cohort in our trial underwent detailed physical examination [12,18].

Potentially the findings from our study are associated with impairments in ß_1_-adrenoreceptors. Poor glycaemic control is associated with chronically elevated catecholamine levels [19] and can induce ß_1_-adrenoreceptor insensitivity. Impairment in ß_1_-adrenoreceptor sensitivity is known to alter the ability of HR to respond adequately to increasing metabolic demands [20]. ß_1_-adrenoreceptors produces positive inotropy, chronotropy and lusitropy with further positive dromotropic effect and pacemaker activity from the sinoatrial node [21]. We hypothesize if ß_1_-adrenoreceptor insensitivity is present, a lower rise in free intracellular Ca^2+^ concentration dysregulates cardiac muscle contraction [22,23] resulting in CI during CPX testing. Taking this into account for our study cohort, we postulate that elevated HbA_1c_ may modify the typical HR response to stress via ß_1_-adrenoreceptor hyposensitivity.

Similar to the findings from our study, previous studies observed reduced cardiac output during sub-maximal exercise intensities in individuals with T1DM [24][13]. Intriguingly, for our data this was not only supported by the decreased ratio of HR at HRTP as percentage of HR_max_, we also found for the non-adjusted stepwise linear regression (data not shown) a significant increased O_2_-Pulse (surrogate parameter for stroke volume) at the HRTP in individuals with poor glycaemic control. We postulate that the decreased ratio of HR at HRTP as percentage of HR_max_ is compensated via increased stroke volume at the HRTP to maintain adequate cardiac output in relation to metabolic demands.

Several studies found a decreased exercise performance in comparison of individuals with T1DM and their healthy counterparts [25][26]. However, little research exists on its relation to glycaemic control [13], and to the best of our knowledge, no trials investigated the influence of CI on exercise performance. As found in our study, CI analyzed via k_HR_ was associated with lowered P_max_ (W.kg^-1^).

ROC curve clearly showed that HbA_1c_ above 7.9% (63 mmol.mol^-1^) was associated with k_HR_ towards CI in the transition of HRTP to P_max_. A low HbA_1c_ accompanied with low risk of hypoglycemic episodes are important aspects of the management of T1DM. However, the percentage of people with T1DM achieving HbA_1c_ within 7.0% (53 mmol.mol^-1^) and 7.5% (58 mmol.mol^-1^) is only from 8% to 28% [27–30] and it is unclear if such glycemic control targets are attainable for most patients. It might be that more applicable HbA_1c_ targets (potentially supported by our threshold of 7.9% (63 mmol.mol^-1^)) accompanied with regular physical activity and exercise could be more beneficial in reduction of risk of all-cause mortality and cardiovascular disease [31] and eventually play a role in restoration of counter-regulatory responses to hypoglycemia [32].

From a clinical point of view the findings from this study could be of immense interest for an exact prescription of exercise intensity. The American Diabetes Association recommends at least 150 min per week of moderate intensity aerobic physical activity, defined as percentages of HR_max_ [33]. In consideration of the results from our study regarding HR at HRTP given as percentages of HR_max_, we might dissuade from using percentages of HR_max_. Fixed percentages of HR_max_ would lead to an overestimation of exercise intensity in individuals with poor glycaemic control as in these patients the anaerobic threshold (HRTP) was found in a lower percentage to HR_max_.

This study is somewhat limited by possible differences in c-peptide status, which was not measured for the purpose of this study. Further studies are needed to investigate k_HR_ and ß_1_-adrenoreceptor sensitivity in people with T1DM.

## Conclusions

This is the first study, which found associations between k_HR_ and HbA_1c_, age and duration of diabetes in people with T1DM. Individuals with poor glycaemic control showed slower increases in HR during early stages of CPX testing, which translated to (i) a decreased ratio of HR at HRTP as percentage of HR_max_ and (ii) a lowered body weight-relativized P_max_. Age and diabetes duration were also found to play a role for these findings. However, both factors contributed minimally to the results (age: *β = -0*.*23; diabetes duration*: *β = 0*.*20)*.

## Supporting information

S1 Checklist Trend statement checklist. (PDF)

S1 Text Study protocol. (PDF)
