# Supplementary material for: Heart rate dynamics during cardio-pulmonary exercise testing are associated with glycemic control in individuals with type 1 diabetes
Source: PLoS One. 2018 Apr 2;13(4):e0194750. doi: 10.1371/journal.pone.0194750 (PMC5880363; doi:10.1371/journal.pone.0194750)
Supplement: S1 Text — (PDF) [file pone.0194750.s002.pdf]

Protocol

Trial ID: NN1250-3999

A trial comparing the effect of exercise on blood glucose between insulin degludec and insulin glargine in subjects with type 1 diabetes

Including:

Substantial protocol amendment no 1, version 1.0, 24 January 2013

Redacted protocol
Includes redaction of personal identifiable information only.

Trial phase: 1

.

Protocol originator:
[redacted]
Clinical Pharmacology - Diabetes

This confidential document is the property of Novo Nordisk. No unpublished information contained herein may be disclosed without prior written approval from Novo Nordisk. Access to this document must be restricted to relevant parties.

Table of contents

Page

Table of contents **2**

Table of figures **6**

Table of tables **6**

List of abbreviations **7**

1 Summary **9**

2 Flow chart **11**

3 Background information and rationale for the trial **17**
3.1 Rationale for the trial 17

4 Objectives and endpoints **19**
4.1 Objectives 19
4.1.1 Primary objective 19
4.1.2 Secondary objectives 19
4.2 Endpoints 19
4.2.1 Primary endpoint 19
4.2.2 Secondary endpoints 19
4.2.2.1 Pharmacodynamic endpoints 19

5 Trial design **21**
5.1 Type of trial 21
5.2 Rationale for trial design 22
5.3 Treatment of subjects 23
5.4 Rationale for treatment 24

6 Trial population **25**
6.1 Number of subjects 25
6.2 Inclusion criteria 25
6.3 Exclusion criteria 25
6.4 Withdrawal criteria 27
6.5 Run-in period exclusion criteria 27
6.6 Steady state period exclusion criteria 28
6.7 Subject replacement 29
6.8 Rationale for trial population 29

7 Trial schedule **30**

8 Methods and assessments **31**
8.1 Visit procedures 31
8.1.1 Visit schedule 32
8.1.2 Before screening 32
8.1.3 Screening visit (Visit 1) 32
8.1.4 Visits 2 and 7 (run-in period) 34
8.1.5 Visits 3–5, Visits 8–10 and unscheduled telephone visits (run-in period) 36
8.1.6 Visits 6 and 11 (steady state period) 36

8.1.6.1 Day 1–3 of V6 and V11 36
8.1.6.2 Day 4 of V6 and V11 37
8.1.6.3 Day 5 of V6 and V11 (exercise bout day)....................38
8.1.6.4 Day 6 of V6 and V11 39
8.1.7 Follow-up visit (Visit 12) 39
8.2 Concomitant illness and medical history....................40
8.3 Concomitant medication....................40
8.4 Assessments for pharmacodynamics 41
8.4.1 Blood glucose measurement....................41
8.4.1.1 Rescue criteria for hypo- and hyperglycaemic episodes during and after exercise....................41
8.4.1.2 Carbohydrate administration....................41
8.4.2 Continuous glucose monitoring (CGM) 42
8.4.3 Exercise bout....................42
8.4.4 Counter-regulatory hormones....................42
8.4.5 Beta-hydroxybutyrate 43
8.5 Assessments for safety....................43
8.5.1 Physical examination....................43
8.5.2 Vital signs....................43
8.5.3 Electrocardiogram (ECG)....................44
8.5.4 Self measured plasma glucose (SMPG)....................44
8.5.5 Hypoglycaemic episodes 45
8.5.6 ADA classification of hypoglycaemia....................45
8.5.7 Additional definitions of hypoglycaemia....................46
8.5.8 Adverse events....................47
8.5.8.1 Adverse events describing injection site reactions....................47
8.5.9 Laboratory assessments 47
8.5.9.1 HbA_{1c} and fasting C-peptide....................48
8.5.9.2 Haematology 48
8.5.9.3 Biochemistry 48
8.5.9.4 Coagulation 49
8.5.9.5 Urinalysis 49
8.5.9.6 Hepatitis B and C and HIV 49
8.5.9.7 Pregnancy test 49
8.5.10 Alcohol breath test and screen for drugs....................49
8.6 Other assessments....................50
8.6.1 Diagnosis of diabetes....................50
8.6.2 Demography....................50
8.6.3 Body measurements....................50
8.6.4 IPAQ....................50
8.6.5 Indirect calorimetry....................50
8.6.6 VO_{2peak} test....................50
8.7 Volume of blood to be drawn during the trial....................51
8.8 Subject compliance....................51

9 Trial supplies 52
9.1 Trial products....................52
9.2 Non-investigational medicinal products 52

9.3 Labelling 52
9.4 Storage, accountability and destruction 52
9.5 Auxiliary supply 54

10 Randomisation procedure 55

11 Adverse events, technical complaints and pregnancies 56
11.1 Definitions 56
11.2 Reporting of adverse events 59
11.3 Follow-up of adverse events 60
11.4 Technical complaints and technical complaint samples 61
11.4.1 Reporting of technical complaints 61
11.4.2 Collection, storage and shipment of technical complaint samples 61
11.5 Pregnancies 61
11.6 Precautions and/or overdose 62
11.7 Committees related to safety 63
11.7.1 Novo Nordisk safety committee 63

12 Case report forms 64
12.1 Corrections to case report forms 64
12.2 Case report form flow 64

13 Monitoring procedures 66

14 Data management 67

15 Computerised systems 68

16 Statistical considerations 69
16.1 Sample size calculation 69
16.2 Definition of analysis sets 70
16.3 Primary endpoint 71
16.3.1 Analysis of primary endpoint 71
16.4 Secondary endpoints 71
16.4.1 Secondary pharmacodynamic endpoints 71
16.4.2 Analysis of secondary pharmacodynamic endpoints 73
16.4.3 Other pharmacodynamic parameters and measurements 73
16.4.4 Analysis of other pharmacodynamic parameters and measurements 74
16.4.5 Safety parameters 74

17 Ethics 76
17.1 Informed consent 76
17.2 Data handling 77
17.3 Premature termination of the trial and/or trial site 77

18 Protocol compliance 78

19 Audits and inspections 79

20 Critical documents 80

21 Responsibilities 81

22 Reports and publications 84
22.1 Communication of results 84

22.1.1 Authorship 85
22.2 Investigator access to data and review of results 85
23 Retention of clinical trial documentation 86
24 Institutional Review Boards/Independent Ethics Committees and regulatory authorities 87
25 Indemnity statement 88
26 References 89

Attachment I – Global List of key staff and relevant departments and CRO

Table of figures

Page

Figure 5-1 Schematic diagram of the trial design....21
Figure 8-1 ADA classification of hypoglycaemia....46

Table of tables

Page

Table 2-1 Trial flow chart....11
Table 2-2 Exercise bout visit schedule (Day 5-6 of Visits 6 and 11)....15
Table 5-1 IDeg and IGlar titration algorithm....23
Table 16-1 Sample size calculation....70
Table 16-2 Overview of pharmacodynamic endpoints for exercise period....71
Table 16-3 Overview of pharmacodynamic endpoints for period following exercise....72
Table 16-4 Overview of other pharmacodynamic parameters....73

List of abbreviations

ADA	American Diabetes Association
AE(s)	adverse event(s)
ALAT	alanine aminotransferase
ALP	alkaline phosphatase
APTT	activated partial thromboplastin
ASAT	aspartate aminotransferase
BG	blood glucose
BMI	body mass index
CGM	continuous glucose monitoring
CHO	carbohydrate (carbon hydrogen oxygen)
CLAE	clinical laboratory adverse event
CNS	central nervous system
CRF	case report form
CRO	contract research organisation
CSII	continuous subcutaneous insulin infusion
CTA	clinical trial application
CTR	clinical trial report
DBL	database lock
DCF	data clarification form
DUN	dispensing unit number
ECG	electrocardiogram
EXE	exercise
FAS	full analysis set
FDAAA	Food and Drug Administration Amendments Act
GCP	Good Clinical Practice
GGT	gamma-glutamyltransferase
HbA_{1C}	glycosylated haemoglobin
HBsAg	hepatitis B surface antigen
HIV	human immunodeficiency virus
IAsp	insulin aspart
ICMJE	International Committee of Medical Journal Editors
IDeg	insulin degludec

i.v.	intravenous
IEC	independent ethics committee
IG	interstitial glucose
IGlar	insulin glargine
IMP	investigational medicinal product
INR	international normalised ratio
IPAQ	International Physical Activity Questionnaire
IRB	Institutional Review Board
MCH	mean corpuscular haemoglobin
MCHC	mean corpuscular haemoglobin concentration
MCV	mean corpuscular volume
MESI(s)	medical event(s) of special interest
MET	metabolic equivalent of task
NIMP	non-investigational medicinal product
NPH	Neutral Protamine Hagedorn
NYHA	New York Heart Association
OC	oracle clinical
PD	pharmacodynamic(s)
PG	plasma glucose
SAE	serious adverse event
SAP	statistical analysis plan
s.c.	subcutaneous(ly)
SMPG	self-measured plasma glucose
StD	standard deviation
SUSAR	suspected unexpected serious adverse reaction
TMM	trial materials manual
ULN	upper limit of normal
UTN	Universal Trial Number
VO_{2peak}	peak rate of oxygen uptake

1 Summary

Objectives and endpoints

Primary objective

- To compare changes in blood glucose during exercise in subjects with type 1 diabetes treated with insulin degludec (IDeg) or insulin glargine (IGlar).

Secondary objectives

- To compare changes in blood glucose after exercise in subjects with type 1 diabetes treated with IDeg or IGlar.
- To compare the risk of hypoglycaemia during and after exercise in subjects with type 1 diabetes treated with IDeg or IGlar.

Primary endpoint

- $BG_{pre\text{-}exe}$ - $BG_{minimum,exe}$, difference between blood glucose concentration before exercise and the minimum blood glucose concentration observed during exercise (from 0 to 30 minutes).

Key secondary endpoints

- $BG_{mean,exe}$, mean blood glucose concentration during exercise (from 0 to 30 minutes).
- $BG_{mean,30–180min,post\text{-}exe}$, mean blood glucose concentration between 30 and 180 minutes, i.e. post-exercise.
- $BG_{minimum,30–180min,post\text{-}exe}$, minimum blood glucose concentration between 30 and 180 minutes, i.e. post-exercise.

Trial design

This is a single-centre, randomised, open-label, two-period, multiple-dose, cross-over trial comparing the changes in blood glucose and risk of hypoglycaemia during and after exercise in subjects with type 1 diabetes treated with IDeg or IGlar.

Trial population

Forty (40) male and female subjects with type 1 diabetes will be randomised in the trial.

Key Inclusion Criteria

- Male or female aged 18–45 years (both inclusive).
- Type 1 diabetes mellitus (as diagnosed clinically) ≥ 12 months.
- Body mass index 18.0–27.0 kg/m^2 (both inclusive).

- Subjects performing regular physical cardiorespiratory activity.
- Glycosylated haemoglobin (HbA_{1c}) ≤ 9.5 %.

Key Exclusion Criteria

- Subject who has donated any blood or plasma in the past month or more than 500 mL within 3 months prior to screening.
- Smoker (defined as a subject who is smoking more than 5 cigarettes or the equivalent per day).
- Not able or willing to refrain from smoking and use of nicotine substitute products during the inpatient period.
- Supine blood pressure at screening (after resting for at least 5 minutes) outside the range of 90–140 mmHg for systolic or 50–90 mmHg for diastolic.

Assessments

Blood glucose concentration will be measured frequently during a 30-minutes exercise bout and for the following two and a half hour period. Number of carbohydrate administrations and minor and severe hypoglycaemic episodes will also be recorded during and after exercise (for 24 hours after start of exercise).

Trial products

The following investigational medicinal products (IMPs) will be used:

- Insulin degludec (100 U/mL) in 3 mL pre-filled investigational pens (PDS290).
- Insulin glargine (Lantus®), 100 U/mL in 3 mL SoloStar®.

During each treatment period each subject will be treated pre-breakfast with once-daily injections of either IMP at an individual dose level. The IMPs will be administered subcutaneously into a lifted skin fold on the anterior surface of the thigh.

2 Flow chart

Table 2-1 Trial flow chart

Trial Periods	Screening	Treatment period 1						Treatment period 2						Follow-up
		Run-in period (14-28 days)		Steady state period (6 days)				Run-in period (14-28 days)		Steady state period (6 days)				
**Visit number**	**1**	**2**	**3-5[a]**	**6**				**7**	**8-10[a]**	**11**				**12**
**Time of visit**	2-21 days before V2	2-21 days after V1		V6 Day 1-3[b]	V6 Day 4	V6 Day 5	V6 Day 6	7-21 days after V6 Day 6		V11 Day 1-3[b]	V11 Day 4	V11 Day 5	V11 Day 6	7-21 days after V11 Day 6
**SUBJECT RELATED INFO/ASSESSMENTS**														
**Informed consent**	x[1]													
**In/exclusion criteria**	x	x[2]												
**Dosing day exclusion criteria**		x[3]	x[3]	x[4]	x[4]	x[4]	x[4]	x[3]	x[3]	x[4]	x[4]	x[4]	x[4]	
**Randomisation**		x												
**Withdrawal criteria**		x	x	x	x	x	x	x	x	x	x	x	x	
**Alcohol breath test**	x	x			x			x			x			
**Concomitant illness**	x													
**Concomitant medication[5]**	x	x	x	x	x	x	x	x	x	x	x	x	x	x
**Demography**	x													
**Body measurements**	x					x[6]						x[6]		x[6]
**Diagnosis of diabetes**	x													
**Medical history**	x													
**EFFICACY**														
**CGM**					x	x	x				x	x	x	
**Glucose**						x	x					x	x	
**Counter-regulatory hormones**						x						x		

**Trial Periods**	**Screening**	**Treatment period 1**						**Treatment period 2**						**Follow-up**
		**Run-in period** (14-28 days)		**Steady state period** (6 days)				**Run-in period** (14-28 days)		**Steady state period** (6 days)				
**Visit number**	**1**	**2**	**3-5**[a]	**6**				**7**	**8-10**[a]	**11**				**12**
**Time of visit**	2-21 days before V2	2-21 days after V1		V6 Day 1-3[b]	V6 Day 4	V6 Day 5	V6 Day 6	7-21 days after V6 Day 6		V11 Day 1-3[b]	V11 Day 4	V11 Day 5	V11 Day 6	7-21 days after V11 Day 6
**Beta-hydroxybutyrate**						x						x		
**SAFETY**														
**Adverse events**		x	x	x	x	x	x	x	x	x	x	x	x	x
**Hypoglycaemic episodes**		x	x	x	x	x	x	x	x	x	x	x	x	x
**ECG**	x													x
**Physical examination**	x					x						x		x
**Vital signs**	x	x				x		x				x		x
**Biochemistry**	x													x
**Coagulation parameters**	x													
**HbA_{1c}**	x							x						
**Fasting C-peptide**		x												
**Haematology**	x													x
**Hepatitis**	x													
**HIV**	x													
**Pregnancy test**[7]	x	x			x			x			x			x
**Urinalysis**	x													x
**Screen for drugs**	x													
**OTHER ASSESSMENTS**														
**Physical activity**		x	x	x	x	x	x	x	x	x	x	x	x	
**IPAQ**[8]	x													
**SMPG**[9]		x	x	x	x			x	x	x	x			
**Indirect calorimetry**	x					x						x		
**VO_{2peak} test**	x													
**Carbohydrate admin.**[11]						x	x					x	x	

**Trial Periods**	**Scree ning**	**Treatment period 1**						**Treatment period 2**						**Follow-up**
		**Run-in period** (14-28 days)		**Steady state period** (6 days)				**Run-in period** (14-28 days)		**Steady state period** (6 days)				
**Visit number**	**1**	**2**	**3-5**[a]	**6**				**7**	**8-10** [a]	**11**				**12**
**Time of visit**	2-21 days before V2	2-21 days after V1		V6 Day 1-3 [b]	V6 Day 4	V6 Day 5	V6 Day 6	7-21 days after V6 Day 6		V11 Day 1-3 [b]	V11 Day 4	V11 Day 5	V11 Day 6	7-21 days after V11 Day 6
**TRIAL MATERIAL**														
**Administration of trial product (IMPs)**		x	x	x	x	x	x	x	x	x	x	x	x	
**Drug accountability**					x	x	x				x	x	x	
**REMINDERS**														
**Hand out diary**	x													
**Attend visit fasting**		x												
**Dispense trial product**		x						x						
**Supply blood glucose meter**		x												
**Self-administered bolus insulin**[9]		x	x	x	x	x	x	x	x	x	x	x	x	
**Clinical trial unit admission**					x	x	x				x	x	x	
**Exercise bout**[10]						x						x		
**Affirmation statement**														x
**End of trial**														x

[a] Visits conducted by telephone at least once the first week and at least twice/week the following weeks.
[b] Visits conducted by telephone on each day during Day 1-3 of the steady state periods.
[1]Check of signed and dated informed consent prior to screening visit. At an information meeting held prior to the screening visit, potential subjects will be provided with oral and written information about the trial and the procedures involved. The informed consent procedure must be completed before any trial related activities take place.
[2]Confirmation that the subject is eligible for continuation in the trial.
[3]Run-in period exclusion criteria to be checked.
[4] Steady state period exclusion criteria to be checked.
[5]Including current insulin treatment.
[6]Only body weight
[7]For women only. A blood pregnancy test will be performed at screening and follow-up. During the rest of the trial a urine stick test will be performed.
[8]Short version of International Physical Activity Questionnaire (IPAQ) modified to cover physical activity during the last three months.
[9]During the clinical unit admission, the diary will not be used, as self-measured plasma glucose (SMPG) and self-administered bolus insulin will be captured by the site staff in the subject’s medical record.
[10]Exercise for 30 minutes to be performed on a cycle ergometer.
[11]Type, amount and time of carbohydrate administration to be recorded in the case report form (CRF).

Table 2-2 Exercise bout visit schedule (Day 5-6 of Visits 6 and 11)

Seq. no.	Nominal time		Day no.	Scheduled time	Intervention	Parameter	Assessment		
	Hours	Minutes				Blood Glucose	Carbohydrate administration	Counter-reg. hormones	Beta-hydroxy butyrate
1	-3	00	5	12:00	Standardised meal. Insulin aspart (IAsp) dose reduced by 50% compared with the usual IAsp dose.				
2	-1	00	5	14:00		x			
3	-0	10	5	14:50	Indirect calorimetry (baseline)	x[1]			
4	0	00	5	15:00		x[1,2]		x[2]	x[2]
5	0	00	5	15:00	Start of exercise bout and indirect calorimetry	Every 5 min	As and when required[3]		
6	0	30	5	15:30	End of exercise bout and indirect calorimetry			x	x
7	0	40	5	15:40		Every 10 min[4]			
8	1	00	5	16:00				x	x
9	2	00	5	17:00				x	x
10	3	00	5	18:00	Standardised meal			x[5]	x[5]
11	4	30	5	19:30		x[6]			
12	7	00	5	22:00		x			
13	13	00	6	04:00		x			
14	16	00	6	07:00	IMP administration and standardised meal	x[5]			
15	17	30	6	08:30		x[6]			
16	21	00	6	12:00	Standardised meal	x[5]			
17	22	30	6	13:30		x[6]			
18	24	00	6	15:00	End of CGM and discharge from the clinical unit				

Note – time stated (as 24 hour clock) is provided as a guide only. Test day activities will be performed in accordance with the nominal times stated.

[1] The mean of blood glucose (BG) values taken 10 min prior and immediately prior to exercise bout should be used as the pre-exercise blood glucose value to check the steady state exclusion criteria.

[2] Immediately prior to exercise bout

[3] If BG < 2.8 mmol/L or plasma glucose (PG) < 3.1 mmol/L a hypoglycaemic episode will be reported and oral carbohydrates will be provided and recorded (type, amount and time).

[4] The 180 min BG sample should be taken before the standardised meal

[5] Before the standardised meal

[6] 90 min after the start of the standardised meal

3 Background information and rationale for the trial

In this document, the term investigator refers to the individual responsible for the overall conduct of the clinical trial at a trial site.

Improvement in long-term glucose control can reduce the incidence of complications and delay the progression of existing complications in type 1 and type 2 diabetes[1,2]. In general, it is recommended to aim for an glycosylated haemoglobin (HbA_{1c}) < 7.0%, without significant episodes of hypoglycaemia. Insulin analogues have been developed to more closely mimic the pharmacokinetics of endogenous insulin secretion as compared with human insulin preparations and are now an established part of diabetes management.

Insulin degludec (IDeg) is an ultra-long-acting basal insulin that offers a flat, stable steady state time-action profile yielding stable 24-hour basal control of blood glucose concentration for all subjects with diabetes mellitus. IDeg is under development both as a new generation basal insulin analogue (Insulin degludec; IDeg) and in combination with the marketed, rapid-acting insulin analogue, insulin aspart (insulin degludec/insulin aspart, IDegAsp).

IDeg is modified such that the amino acid residue threonine in position B30 of human insulin has been omitted and the ε-amino group of lysine in position B29 has been coupled to hexadecanedioic acid via a glutamic acid spacer. This structure allows IDeg to form soluble and stable multi-hexamers, resulting in a depot in the subcutaneous (s.c.) tissue after injection.

The gradual separation of IDeg monomers from the multi-hexamers results in a slow and continuous delivery of IDeg from the s.c. injection site into the circulation, leading to the observed ultra-long pharmacokinetic and pharmacodynamic profiles. Furthermore, binding of the fatty acid moiety of IDeg to albumin contributes to some extent to the protraction mechanism. At the target tissues, IDeg monomers bind to and activate insulin receptors triggering the same cellular effects as human insulin such as promoting glucose uptake.

IDeg is efficacious in terms of lowering HbA_{1c} and results in a lower risk of nocturnal hypoglycaemia compared to current basal insulin products due to its distinct pharmacological profile. Detailed information is available in the current edition of the investigators brochure for IDeg[3]. IDeg has been shown to be well tolerated with an adverse event (AE) profile similar to that of other marketed insulin products and no unexpected findings or unacceptable risks have been identified.

3.1 Rationale for the trial

Physical activity has an important role in the treatment of both type 1 and type 2 diabetes, and regular exercise is a recommended part of diabetes patients' lifestyle[4,5]. For people with diabetes, exercise improves metabolic control, decreases exogenous insulin requirements by increasing

insulin sensitivity and may prevent or postpone diabetes-related complications[6,7]. Among diabetes patients, hypoglycaemia during or after exercise is the most common risk associated with exercise. It would therefore be important to learn more about this risk when people with diabetes are treated with basal insulin analogues such as IDeg and insulin glargine (IGlar). The main purpose of this trial is to compare changes in blood glucose and risk of hypoglycaemia during and after exercise in subjects with type 1 diabetes treated with IDeg or IGlar.

4 Objectives and endpoints

4.1 Objectives

4.1.1 Primary objective

- To compare changes in blood glucose during exercise in subjects with type 1 diabetes treated with IDeg or IGlar.

4.1.2 Secondary objectives

- To compare changes in blood glucose after exercise in subjects with type 1 diabetes treated with IDeg or IGlar.
- To compare the risk of hypoglycaemia during and after exercise in subjects with type 1 diabetes treated with IDeg or IGlar.

4.2 Endpoints

4.2.1 Primary endpoint

- $BG_{pre\text{-}exe} - BG_{minimum,exe}$, difference between blood glucose concentration before exercise and the minimum blood glucose concentration observed during exercise (from 0 to 30 minutes).

4.2.2 Secondary endpoints

4.2.2.1 Pharmacodynamic endpoints

The following endpoints will be derived for the exercise period:

- $BG_{mean,exe}$, mean blood glucose concentration during exercise (from 0 to 30 minutes).
- $BG_{pre\text{-}exe}$ - $BG_{30min,exe}$, difference between blood glucose concentration before exercise and after 30 minutes of exercise.
- $Hypo_{exe}$, number of minor and severe hypoglycaemic episodes during exercise (from 0 to 30 minutes).
- CHO_{exe}, number of carbohydrate administrations given during exercise (from 0 to 30 minutes) in response to hypoglycaemia.

The following endpoints will be derived for the period following exercise:

- $BG_{mean,30\text{–}180min,post\text{-}exe}$, mean blood glucose concentration between 30 and 180 minutes, i.e. post-exercise.
- $BG_{minimum,30\text{–}180min,post\text{-}exe}$, minimum blood glucose concentration between 30 and 180 minutes, i.e. post-exercise.

- $tBG_{minimum,30–180min,post-exe}$, time from end of exercise until minimum blood glucose concentration between 30 and 180 minutes, i.e. post-exercise.
- $Hypo_{24h,post-exe}$, number of minor and severe hypoglycaemic episodes post-exercise (for 24 hours after start of exercise).
- Nocturnal $hypo_{24h,post-exe}$, number of nocturnal minor and severe hypoglycaemic episodes post-exercise (for 24 hours after start of exercise).
- $CHO_{24h,post-exe}$, number of carbohydrate administrations given post-exercise (for 24 hours after start of exercise) in response to hypoglycaemia.

5 Trial design

5.1 Type of trial

This is a single-centre, randomised, open-label, two-period, multiple-dose, cross-over trial comparing the changes in blood glucose and risk of hypoglycaemia during and after exercise in subjects with type 1 diabetes treated with IDeg or IGlar. The overall trial design and visit schedule are outlined in Figure 5-1.

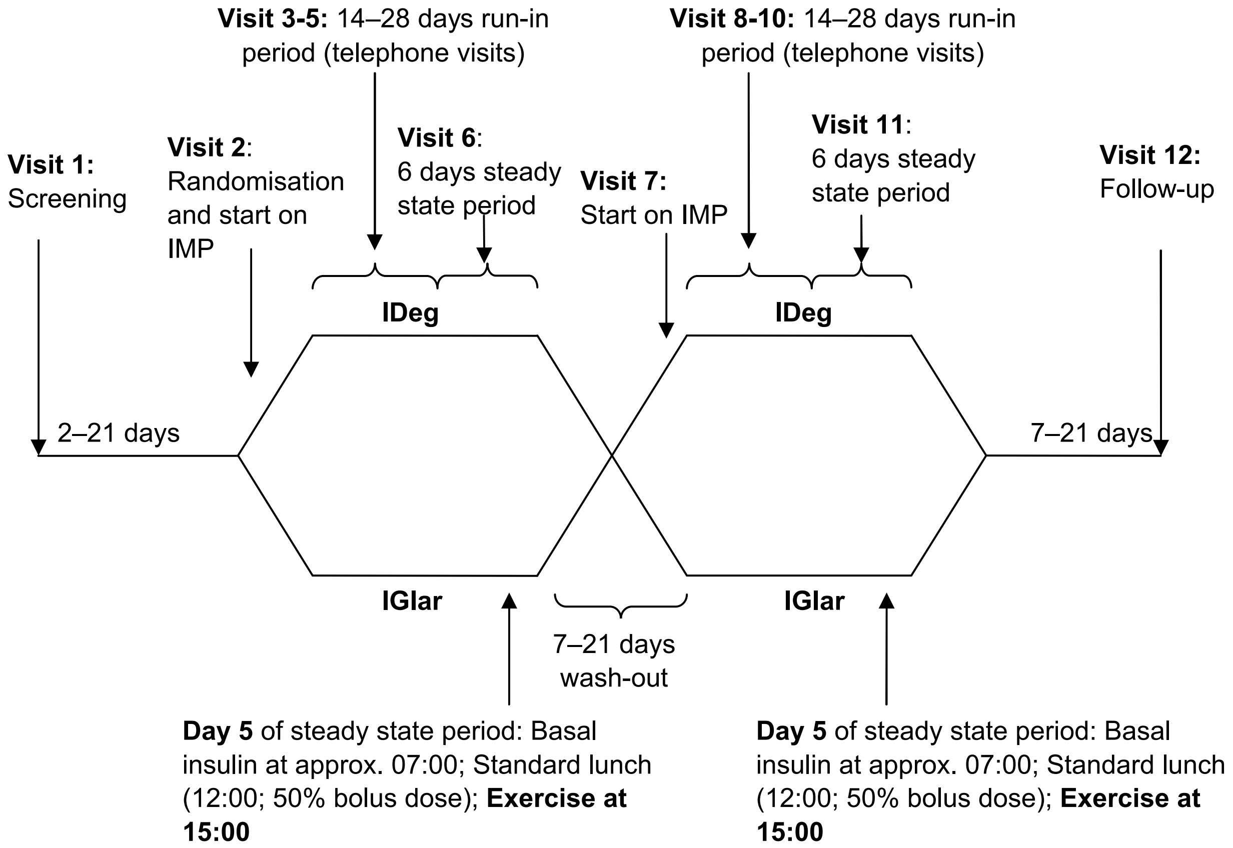

Figure 5-1 Schematic diagram of the trial design.

The trial consists of a screening visit (Visit 1), a randomisation and start on investigational medicinal product (IMP) visit (Visit 2), telephone visits during a 14–28 days run-in period (at least once the first week and at least twice/week the following weeks) (Visits 3–5 and unscheduled telephone visits), a steady state and exercise visit (Visit 6), a second start on IMP visit (Visit 7), telephone visits during a second 14–28 days run-in period (at least once the first week and at least twice/week the following weeks) (Visits 8–10 and unscheduled telephone visits), a second steady

state and exercise visit (Visit 11), and a follow-up visit (Visit 12). The trial will have a duration of 55–148 days for the individual subject.

At screening, each subject will perform an incremental bicycle exercise bout as well as standard screening procedures in order to assess peak rate of oxygen uptake (VO_{2peak}) and trial participation eligibility. Eligible subjects will be randomised to a treatment sequence consisting of two treatment periods in which the subjects will receive IDeg and IGlar, respectively. Each treatment period consists of 14–28 days run-in followed by a 6-days steady state period. During each treatment period, subjects will be administered either IDeg or IGlar once daily pre-breakfast (at approximately 07:00 hours) at an individual dose level. The individual dose level will be established using a defined titration algorithm during the run-in period and kept constant during the 6-days steady state period. Insulin aspart (IAsp) will be used as bolus insulin. The main purpose of the steady state period is to obtain a reproducible blood glucose profile from day to day and thereby to ensure that blood glucose is at a controlled and similar level before the exercise bouts. For this reason the subjects will have standardised meals during the steady state period (composition of meals to be given in a separate meal plan). During the steady state period, the subjects should maintain their usual exercise and physical activity regularity in order to not markedly affect insulin sensitivity due to changes in physical activity pattern. Moreover, the subjects should not perform unaccustomed physical activity/exercise during the 6-days steady state period.

On Day 5 of each steady state period subjects will perform 30 minutes ergometer bicycling at 65% $VO_{2\,peak}$ at 15:00 hours, which is 3 hours after a standardised lunch. IAsp administered at lunch will be reduced by 50% compared with the usual IAsp dose. During and up to 24 hours after the start of the exercise blood glucose will be monitored by a standard laboratory method, continuous glucose monitoring (CGM) will be performed, and hypoglycaemic episodes as well as carbohydrate administration (type, amount and time) will be recorded. Each subject will stay in-house 24 hours prior to the exercise bout and will remain in-house until 24 hours after the exercise bout.

5.2 Rationale for trial design

The cross-over design has been chosen to increase the power and thereby limit the total number of subjects needed. Randomisation is used in order to avoid bias introduced through an association between IMP product allocation order and subject characteristics.

An open-labelled design is needed as the IMPs are self-administered by the subjects.

A time period between each treatment period of at least 7 days is introduced to ensure wash-out from the previous dosing and thereby to avoid any carry-over effect. The length of the wash-out period is considered sufficient based on the half-life of IDeg (25 hours).

5.3 Treatment of subjects

Investigational medicinal product

The following investigational medicinal products (IMPs) will be used:

- Insulin degludec (100 U/mL) in 3 mL pre-filled investigational pens (PDS290).
- Insulin glargine (Lantus®), 100 U/mL in 3 mL SoloStar®.

During each treatment period each subject will be treated pre-breakfast with once-daily injections of either IMP at an individual dose level with no maximum dose specified. Doses should be titrated at each telephone visit during the run-in period using a stepwise titration algorithm according to Table 5-1. During both treatment periods, the insulin dose adjustments should aim at reaching stable glyceamic control and a pre-breakfast SMPG value between 4.0 and 6.0 mmol/L (71–108 mg/dL).

Titration of IDeg and IGlar should be done based on the mean of three pre-breakfast SMPG values (measurements on the two days just prior and on the day of titration).

Starting dose of IDeg or IGlar will be based on the pre-trial basal insulin dose. At the randomisation visit (Visit 2) subjects should transfer their previous dose of basal insulin 1:1 to IDeg or IGlar. However, if pre-trial basal insulin is administered more often than once daily, the initial dose of IDeg or IGlar should be reduced by 20%.

The IDeg and IGlar dose can be changed in multiples of 2 units according to the algorithm in Table 5-1.

Table 5-1 IDeg and IGlar titration algorithm

**Mean pre-breakfast plasma glucose**		**Adjustment of IDeg or IGlar (U)**
mmol/L	mg/dL	
< 3.1	< 56	- 4
3.1–3.9	56–70	- 2
4.0–6.0 = target	71–108 = target	No adjustment
6.1–10.0	108–180	+ 2
10.1–15.0	181–270	+ 4
> 15.0	> 270	+ 6

The IMPs will be administered s.c. into a lifted skin fold on the anterior surface of the thigh. For both IMPs, the injection site should remain within the same anatomical region throughout the trial, but location within the area should be changed for each injection.

The maximum duration of treatment of a single subject, from first IMP administration to last IMP administration, will be 86 days (excluding the 7–21 days wash-out period).

Non-investigational medicinal products

The following non-investigational medicinal product (NIMP) will be used:

- Insulin aspart (NovoRapid®), 100 U/mL in 3 mL FlexPen®.
- Neutral Protamine Hagedorn (NPH) insulin (Prothaphane®), 100 IU/ml in 3 ml FlexPen®.

Dose levels will be individual with no maximum dose specified. Doses are titrated according to PG values. IAsp will be injected s.c.. IAsp given together with lunch on the day of exercise will be reduced to 50% of the subject's normal IAsp dose as defined at the discretion of the investigator based on IAsp doses given during the steady state period.

The following medications should not be used:

- systemic (oral or intravenous (i.v.)) corticosteroids, MAO inhibitors, systemic non-selective or selective beta-blockers, growth hormone, herbal products or non-routine vitamins. Furthermore, thyroid hormones are not allowed unless the use of these has been stable during the past 3 months.

5.4 Rationale for treatment

Both IMPs will be administered s.c., which is the intended route of administration for IDeg and the approved route of administration for IGlar.

Dosage of the trial products will be individual in order to obtain a reproducible blood glucose profile from day to day. The individual dose level will be established using a defined titration guideline during the run-in period and kept constant during the 6-days steady state period.

IGlar has been included as comparator, since this is a commonly used basal insulin analogue already on the market.

The treatment periods will consist of a run-in period to establish the individual dose level of the IMPs and a steady state period where the dose level is kept constant to obtain a reproducible blood glucose profile from day to day and thereby to ensure that blood glucose is at a controlled and similar level before the exercise bouts.

6 Trial population

6.1 Number of subjects

Country planned to participate: Germany

Number of subjects planned to be randomised/started on trial products: 40

Number of subjects expected to complete the trial: 36

6.2 Inclusion criteria

For an eligible subject, all inclusion criteria must be answered "yes".

1. Informed consent obtained before any trial-related activities. Trial-related activities are any procedures that are carried out as part of the trial, including activities to determine suitability for the trial.
2. Male or female aged 18–45 years (both inclusive).
3. Type 1 diabetes mellitus (as diagnosed clinically) ≥ 12 months.
4. Treated with multiple daily insulin injections or continuous subcutaneous insulin infusion (CSII) ≥ 12 months.
5. Body mass index (BMI) 18.0–27.0 kg/m^2 (both inclusive).
6. Mass-specific VO_{2peak} > 35 and < 60 ml O_2/kg body weight/min.
7. Subjects performing regular physical cardiorespiratory activity to achieve an average total energy expenditure of ≥ 500 metabolic equivalent of task (MET)[8,9]-minutes per week during the last 3 months prior to screening.
8. HbA_{1c} ≤ 9.5 %.

6.3 Exclusion criteria

For an eligible subject, all exclusion criteria must be answered "no".

1. Known or suspected hypersensitivity to trial product(s) or related products.
2. Previous participation in this trial. Participation is defined as randomised.
3. Receipt of any IMP within 3 months prior to screening in this trial.
4. Haemoglobin < 8.0 mmol/L (male) or < 6.4 mmol/L (female), total leukocyte count < 3.0 x 10^9/L, thrombocytes <100 x 10^9/L, serum creatinine levels ≥ 126 µmol/L (male) or ≥ 111 µmol/L (female), alanine aminotransferase > 2 x the upper limit of normal (ULN), bilirubin > 3 x ULN, alkaline phosphatase > 2 x ULN.
5. Suffer from or history of a life threatening disease (i.e. cancer judged not to be in full remission except basal cell skin cancer or squamous cell skin cancer), or any clinically significant respiratory, metabolic, renal, hepatic, gastrointestinal, endocrinological (with the exception of

diabetes mellitus and euthyroid struma), haematological, dermatological, venereal, neurological, psychiatric diseases or other major disorders, as judged by the investigator.

6. Subject with a heart rate < 50 beats per minute (bpm) at screening (after resting for at least 5 min in supine position).
7. Cardiac problems defined as decompensated heart failure (New York Heart Association (NYHA) class III and IV)[10] at any time and/or angina pectoris within the last 12 months and/or acute myocardial infarction at any time.
8. Supine blood pressure at screening (after resting for at least 5 min in supine position) outside the range of 90–140 mmHg for systolic or 50–90 mmHg for diastolic (excluding white-coat hypertension; therefore, if a repeated measurement on a second screening visit shows values within the range, the subject can be included in the trial). This exclusion criterion also pertains to subjects being on antihypertensives.
9. Clinically significant abnormal electrocardiogram (ECG) at screening, as judged by the investigator.
10. Any chronic disorder or severe disease which, in the opinion of the investigator might jeopardise subject's safety or compliance with the protocol.
11. Subject known to be positive for Hepatitis B surface antigen (HBsAg) or Hepatitis C antibodies (or diagnosed with active hepatitis) according to local practice.
12. Positive result to the screening test for HIV-1 antibodies, HIV-2 antibodies or HIV-1 antigen according to locally used diagnostic testing.
13. History of multiple and/or severe allergies to drugs or foods or a history of severe anaphylactic reaction.
14. Subject who has donated any blood or plasma in the past month or more than 500 mL within 3 months prior to screening.
15. Surgery or trauma with significant blood loss (more than 500 mL) within the last 3 months prior to screening.
16. Current treatment with systemic (oral or i.v.) corticosteroids, monoamine oxidase (MAO) inhibitors, non-selective or selective beta-blockers, growth hormone, herbal products or non-routine vitamins. Furthermore, thyroid hormones are not allowed unless the use of these has been stable during the past 3 months.
17. Significant history of alcoholism or drug/chemical abuse as per investigator's judgement or a positive result in the urine drug/alcohol screen at the screening visit.
18. Smoker (defined as a subject who is smoking more than 5 cigarettes or the equivalent per day).
19. Not able or willing to refrain from smoking, or use of nicotine substitute products during the inpatient period.
20. Recurrent severe hypoglycaemia (more than 1 severe hypoglycaemic episode during the past 12 months).
21. Hypoglycaemic unawareness as judged by the investigator or hospitalisation for diabetic ketoacidosis during the previous 6 months.

22. Subject with mental incapacity or language barriers precluding adequate understanding or co-operation or who, in the opinion of their general practitioner or the investigator, should not participate in the trial.
23. Potentially non-compliant or uncooperative during the trial, as judged by the investigator.
24. Any condition that would interfere with trial participation or evaluation of results, as judged by the investigator.
25. Female of childbearing potential who is pregnant, breast-feeding or intend to become pregnant or is not using adequate contraceptive methods (adequate contraceptive measures include sterilisation, hormonal intrauterine devices, oral contraceptives, sexual abstinence or vasectomised partner).

6.4 Withdrawal criteria

The subject may withdraw at will at any time.

The subject may be withdrawn from the trial at the discretion of the investigator due to a safety concern or if judged non-compliant with trial procedures.

A subject must be withdrawn if the following applies:
1. AEs which are considered unacceptable by the subject or the investigator.
2. Protocol deviation: If a protocol deviation or concurrent illness occurs, which, in the clinical judgement of the investigator, may invalidate the trial by interfering pharmacokinetically or pharmacodynamically with the trial products, the subject will be withdrawn by the investigator.
3. Pregnancy or intention of becoming pregnant.
4. The subject donates any blood during the trial.

For all prematurely discontinued subjects, the reason for withdrawal will be recorded and the end of trial form completed. If the withdrawal occurs following dosing with the IMPs, the subject will be asked to meet for a complete follow-up examination.

6.5 Run-in period exclusion criteria

The following exclusion criteria pertain to all dosing days during the run-in period. Meeting one of these exclusion criteria will lead to withdrawal from the trial.

1. More than 1 severe hypoglycaemic episode.
2. Confirmed hyperglycaemia (BG ≥ 17.0 mmol/L (306 mg/dL) or PG ≥ 20.4 mmol/L (368 mg/dL)).
3. Any condition that, in the opinion of the investigator, could interfere with insulin pharmacokinetics and/or glucose metabolism or with the ability to complete the exercise bouts.
4. Use of the following: systemic (oral or i.v.) corticosteroids, MAO inhibitors, systemic non-selective or selective beta-blockers, growth hormone, herbal products or non-routine vitamins.

Furthermore, thyroid hormones are not allowed unless the use of these has been stable during the past 3 months.

5. Not in stable glycaemic control on Day 28 of the run-in period at the latest, as judged by the investigator.

6.6 Steady state period exclusion criteria

The following exclusion criteria pertain to the steady state period.

1. Confirmed hyperglycaemia (BG ≥ 17.0 mmol/L (306 mg/dL) or PG ≥ 20.4 mmol/L (368 mg/dL)).
2. Any condition that, in the opinion of the investigator, could interfere with insulin pharmacokinetics and/or glucose metabolism or with the ability to complete the exercise bouts.
3. Use of the following: systemic (oral or i.v.) corticosteroids, MAO inhibitors, systemic non-selective or selective beta-blockers, growth hormone, herbal products or non-routine vitamins. Furthermore, thyroid hormones are not allowed unless the use of these has been stable during the past 3 months.
4. Unaccustomed exercise as judged by the investigator and/or the subject within 120 hours prior to the exercise bout on Day 5.
5. Episode of severe hypoglycaemia occurring within 72 hours prior to the exercise bout on Day 5.
6. Consumption of more than five cups of coffee, tea or beverages containing methylxanthine (theophylline, caffeine or theobromine) within 48 hours prior to the exercise bout on Day 5.
7. Consumption of alcohol within 48 hours prior to the exercise bout on Day 5.
8. Positive result of alcohol breath test within 24 hours prior to the exercise bout on Day 5.
9. Hypoglycaemia (BG < 2.8 mmol/L or PG < 3.1 mmol/L) within 24 hours prior to the exercise bout on Day 5.
10. A pre-exercise blood glucose value at the exercise bout on Day 5 outside the range of ≥ 5.0 mmol/l (90 mg/dL) and ≤ 10.0 mmol/L (180 mg/dL).
11. A pre-exercise blood glucose value at the second exercise bout day (Visit 11, Day 5) more than 2.0 mmol/L (36 mg/dL) above or below the pre-exercise blood glucose value measured at the first exercise bout.
12. Any condition that the investigator feels may interfere with the ability to complete the exercise bouts, collection or evaluation of data.

Subjects who meet steady state period exclusion criteria 1, 2, or 3 will be withdrawn from the trial.

Meeting steady state period exclusion criteria 4–12 will lead to addition of unscheduled dosing days (maximum number of five unscheduled dosing days per subject per treatment period). Subjects who meet a steady state period exclusion criterion after having used all five allowed unscheduled dosing days will be withdrawn from the trial.

Subjects who meet a steady state period exclusion criterion on an exercise day (Visit 6 Day 5, Visit 11 Day 5) can have their exercise bout rescheduled four times within each treatment period. Remaining unscheduled dosing days may be used if the exercise bout visit cannot be rescheduled within 24 hours.

6.7 Subject replacement

A total of 36 subjects are needed in order to perform the statistical analysis with sufficient power (see Section 16.1). To account for dropouts, a total of 40 subjects will be randomised. Subjects will be replaced to ensure that 36 subjects complete the trial. A replacement subject will be assigned to the same treatment sequence as the subject being replaced (see Section 10).

6.8 Rationale for trial population

The inclusion and exclusion criteria limit the trial population to being in good glycaemic control. This is to recruit subjects being able to achieve the sufficient glycaemic control during the run-in titration period. Moreover, subjects with a history of frequent hypoglycaemic events or hypoglycaemia unawareness will be excluded due to the increased risk of hypoglycaemia during and after physical exercise.

The subjects should be younger to middle-aged adults, who perform regular physical exercise. This is to ensure that the subjects are able to complete the two ergometer bicycling exercise bouts in a safe manner and at the planned exercise intensity. Moreover, the inclusion and exclusion criteria limit the trial population to being of good health and thereby not suffering from any underlying diseases other than type 1 diabetes. This is to avoid compromising the safety of the subjects participating in the trial and to reduce bias on conclusions regarding pharmacodynamics.

7 Trial schedule

Planned duration of recruitment period (i.e. first subject first visit (FSFV) – last subject last visit (LSFV)): 11 weeks

End of trial is defined as LSLV.

Planned date for FSFV: 17-Oct-2012

Planned date for LSLV: 08-Mar-2013

Information of the trial will be disclosed at clinicaltrials.gov and novonordisk-trials.com. According to the Novo Nordisk Code of Conduct for Clinical Trial Disclosure, it will also be disclosed according to other requirements such as those of the International Committee of Medical Journal Editors (ICMJE)[11], the Food and Drug Administration Amendment Act (FDAAA)[12], European Commission Regulation for EudraCT[13] and other relevant recommendations or regulations. If a patient requests to be included in the trial via the Novo Nordisk e-mail contact at these web sites, Novo Nordisk may disclose the investigator's contact details to the patient.

8 Methods and assessments

8.1 Visit procedures

The trial consists of a screening visit (Visit 1), a randomisation and start on IMP visit (Visit 2), telephone visits during a 14–28 days run-in period (at least once the first week and at least twice/week the following weeks) (Visits 3–5 and unscheduled telephone visits), a steady state and exercise visit (Visit 6), a second start on IMP visit (Visit 7), telephone visits during a second 14–28 days run-in period (at least once the first week and at least twice/week the following weeks) (Visits 8–10 and unscheduled telephone visits), a second steady state and exercise visit (Visit 11), and a follow-up visit (Visit 12).

Subjects enrolled in the trial will be provided with a Subject Identification Card (ID card), stating that the subject is participating in the trial and whom to contact (site address, investigator name and telephone number). The subjects should be instructed to return the ID card to the investigator at the last visit or to destroy the card after the last visit.

The investigator must keep a subject screening log and a subject enrolment log. These can be combined in one document.

If a subject is withdrawn from the trial, the investigator must aim to undertake procedures similar to those for Visit 12 (follow-up visit) as soon as possible, if possible. The end of trial form must be completed, and final drug accountability must be performed even if the subject is not able to come to the site. The affirmation statement must be signed.

Although a subject is not obliged to give his/her reason(s) for withdrawing from a trial, the investigator should make a reasonable effort to ascertain the reason(s), while fully respecting the subject’s rights. Where the reasons are obtained, the primary reason(s) (AE, noncompliance with protocol or other) for discontinuation must be specified in the case report form (CRF).

Trial product must not be dispensed to any person not included in the trial.

Review of diaries, laboratory reports, ECGs and IPAQ must be documented either on the front page of the documents and/or in the subject’s medical record. The documents must be reviewed by the investigator to ensure that AEs including any overall change in health and concomitant medication, are reported. If clarification of entries or discrepancies in the diary or IPAQ is needed, the subject should be questioned and a conclusion made in the medical record. Care should be taken not to bias the subject.

For screening failures the screening failure form must be completed with the reason for not continuing in the trial. Serious and non-serious AEs from screening failures must be transcribed by

the investigator into the CRF. Follow-up of serious adverse events (SAEs) should be carried out according to section 11.3.

8.1.1 Visit schedule

A tabulated flow chart covering all scheduled visits is provided in Table 2-1, and a sampling scheme covering Day 5–6 of the steady state visits (Visits 6 and 11) is provided in Table 2-2.

If a subject attends the clinic for an unscheduled visit, the unscheduled visit form must be completed unless the subject attends the clinic to obtain additional medication. In this case, the subject's medical record should be updated accordingly.

8.1.2 Before screening

At an information meeting held prior to the screening visit, potential subjects will be provided with oral and written information about the trial and the procedures involved, in accordance with local requirements. Subjects will be fully informed of their responsibilities and their rights while participating in the trial and of all the procedures involved in the trial as well as the possible risks and benefits of participation in the trial. Subjects will have the opportunity to ask questions and they will have ample time to consider participation. If the subjects wish to participate in the trial, they will be asked to personally sign and date an informed consent form prior to any trial related activities. Likewise, the person who seeks the informed consent must also sign the informed consent form prior to any trial related activities. All subjects will be provided with a copy of their own signed and dated informed consent form.

8.1.3 Screening visit (Visit 1)

The screening will take place 2–21 days prior to the first treatment visit (Visit 2). Subjects will receive a subject number in ascending order.

At the screening visit, the following will be assessed and recorded in the CRF:

- Check of signed and dated informed consent
- Assessment of inclusion and exclusion criteria
- Demography
- Diagnosis of diabetes
- Medical history (any relevant illness in the past)
- Concomitant illness
- Concomitant medication (including current insulin therapy)
- Body measurements
- Physical examination
- Vital signs
- ECG

- Alcohol breath test
- Screen for drugs
- Laboratory examination for Hepatitis B antigen and Hepatitis C antibodies
- Laboratory examination for HIV antibodies and antigen
- Laboratory examination of HbA_{1c}
- Laboratory examination of blood/urine samples for safety assessment (haematology, biochemistry, coagulation and urinalysis)
- Pregnancy test (in women)
- Result from IPAQ
- Result from VO_{2peak} test

The investigator will review all information obtained from the screening procedures. Subjects who comply with all the inclusion and exclusion criteria will be invited for the first treatment visit (Visit 2).

Screened subjects who do not comply with all inclusion and exclusion criteria are excluded, and their data will be recorded on a screening failure log to be kept at the trial site. For screening failures the screening failure form must be completed with the reason for not continuing in the trial. Serious and non-serious AEs from screening failures must be transcribed by the investigator into the CRF. Follow-up of SAEs should be carried out according to section 11.3.

The investigator must provide information to the subjects on insulin treatment for the day before Visits 2 and 7 and provide NPH insulin (if needed). Subjects receiving at least a once-daily injection of a long-acting insulin product should not inject any long-acting insulin within 24 hours before Visits 2 and 7 (i.e. no later than approximately at 07:00 hours on the day before dosing). Subjects that normally dose long-acting insulin after 07:00 hours in the morning should instead switch to NPH insulin on the day before Visits 2 and 7.

The last injection of NPH insulin and other intermediate-acting insulin products should be done no later than 10 hours before dosing on Visits 2 and 7 (i.e. no later than approximately at 21:00 hours on the day before dosing). On the days of Visits 2 and 7, no insulin should be injected prior to the visit. Subjects on CSII do not need to switch to NPH insulin, but should arrive at the first dosing visit with their basal rate running.

Subjects will be provided with a diary and instructed on how to record the following during the treatment periods:
- Self Measured Plasma Glucose (SMPG) values
- Changes in concomitant medication
- Daily physical activity

- Insulin treatment (name, dose, date and time)
- AEs

SMPG values and information on daily physical activity will be used as help for the investigator during titration of the individual insulin doses. SMPG values taken during the steady-state periods should be transcribed to the CRF.

SMPG values, changes in concomitant medication and insulin treatment should also be recorded for the 48 hours leading up to Visits 2 and 7. The subjects should be supplied with a blood glucose (BG) meter, lancets, test strips and control solution. The investigator must ensure that all BG meters use plasma calibrated strips for SMPG. Instruct the subject in conducting SMPG and how to use the BG meter, including regular calibration according to the manufacturer's instruction.

Subjects will be informed:

- That they should pay special attention to any symptoms of hypoglycaemia on the day of the VO_{2peak} test (after the test) and conduct SMPG if a hypoglycaemic episode is suspected.
- That the frequency and intensity of their usual physical activities/sport should be maintained throughout the trial (in order to minimise significant changes in insulin sensitivity).
- That they should maintain their usual diet during the run-in period.
- That they will be required to standardise their dietary intake in the 6-days steady state period.
- To avoid use of the following products: systemic (oral or i.v.) corticosteroids, monoamine oxidase (MAO) inhibitors, systemic non-selective or selective beta-blockers, growth hormone, non-routine vitamins and herbal products. Furthermore, the use of thyroid hormones must be stable.
- To avoid injection of insulin detemir and IGlar within 24 hours prior to dosing with IMP.
- To avoid injection of intermediate-acting insulin products (e.g. NPH insulin) within 10 hours prior to dosing with IMP.
- To avoid injection of any insulin prior to the visit on the days of Visits 2 and 7.
- That they should attend Visit 2 fasting.

8.1.4 Visits 2 and 7 (run-in period)

The subjects will be asked to attend the clinical unit at approximately 06:30 hours in the morning on Visit 2, in a fasting condition (only water from approximately 21:00 hours the evening before the visit). A rescheduling of the visit (within 1–7 days) is allowed once in case subjects have failed to be fasting. Fasting is not needed before Visit 7.

Results from the screening visit (Visit 1) must be available at Visit 2 and assessed to be acceptable by the investigator. These results must be verified by the signing and dating of test results by the investigator.

The following procedures will be performed:

- Check that all inclusion and exclusion criteria have been assessed and that the subject is eligible to continue in the trial
- Check of withdrawal criteria
- Randomisation (only at Visit 2)
- Check of dosing day exclusion criteria (run-in period exclusion criteria)
- Alcohol breath test
- Check of concomitant medication
- Vital signs
- Laboratory examination of fasting C-peptide (only at Visit 2)
- Urine pregnancy test (in women)
- Laboratory examination of HbA_{1c} (only at Visit 7)

Subjects eligible for trial continuation should take a pre-breakfast SMPG value. Trial product will be self-administrated at approximately 07:00 hours. Breakfast will then be provided.

In addition, the following must be carried out:

- Supply the subjects with trial products and IAsp.
- Give instructions in insulin injection techniques and the use of the insulin pens used in this trial. (The investigator must document that direction for use is given to each subject orally and/or in writing at each dispensing visit.)
- Inform the subjects about trial product storage conditions.
- Provide information to the subjects on insulin treatment during the treatment periods.
- Supply the subjects with dietary guidelines to be followed in the steady state period.
- From the diary, the following information must be transcribed to the CRF:
    - Current diabetes therapy for the 48 hours leading up to the visit (name, dose, date and time)
    - Hypoglycaemic episodes
    - AEs
    - Changes in concomitant medication
- Inform the subjects to contact the investigator if an episode of hyperglycaemia (BG ≥ 17.0 mmol/L (306 mg/dL) or PG ≥ 20.4 mmol/L (368 mg/dL)) occurs. Subjects should also be informed about the consequences of a hyperglycaemic episode (i.e. withdrawal).
- Remind the subjects that they must comply with the requirements given in section 8.1.3 regarding medication, exercise and diet during the run-in period
- Remind the subjects that dosing with the IMPs should occur pre-breakfast, in the morning at approximately 07:00 hours (± 1 hour allowed) throughout the run-in period.

8.1.5 Visits 3–5, Visits 8–10 and unscheduled telephone visits (run-in period)

During the run-in periods, telephone visits will be conducted at least once the first week and at least twice/week the following weeks to assess treatment and adjust dose, if necessary. Scheduled telephone Visits 4–5 and Visits 9–10 are to be used during the second week of the run-in periods. If the run-in periods continue for more than 14 days (to ensure stable glycaemic control), at least two telephone visits/week should be added as unscheduled telephone visits.

The investigator will ask about and check the following (must be transcribed to the CRF at the next visit to the site):

- Withdrawal criteria
- Dosing day exclusion criteria (run-in period exclusion criteria)
- Concomitant medication
- From the diary:
  - Insulin treatment (name, dose, date and time)
  - Hypoglycaemic episodes
  - AEs
  - Changes in concomitant medication

Please refer to section 5.3 for titration guidance of the IMPs to achieve stable glycaemic control during the run-in periods. The subjects should be advised on how to adjust their insulin treatment accordingly. The subjects should self-administer IMP at approximately 07:00 hours (± 1 hour allowed) on each day of the run-in period.

8.1.6 Visits 6 and 11 (steady state period)

During the steady state periods, the current dose of IMP should not be changed. Dosing with IMP should occur pre-breakfast, in the morning at approximately 07:00 hours (± 15 min allowed) throughout the steady state period. The composition of the standardised meals will be given in a separate meal plan (to be filed in the subject’s medical record and the trial investigator file).

8.1.6.1 Day 1–3 of V6 and V11

On Day 1–3 of the steady state periods, a telephone visit should be conducted on each day.

The investigator will ask about and check the following (must be transcribed to the CRF at the next visit to the site):

- Withdrawal criteria
- Dosing day exclusion criteria (steady state period exclusion criteria)
- Concomitant medication
- From the diary:

- Insulin treatment (name, dose, date and time)
- Hypoglycaemic episodes
- AEs
- Changes in concomitant medication
- SMPG values

The subject should be reminded to:

- Not to change the current dose of the IMP during the steady state period.
- Comply with IMP dosing at 07:00 hours ± 15 min.
- Comply with the requirements given in section 6.6 regarding medication, exercise and beverage intake during the steady state period.
- Measure PG 3 hours after lunch (in addition to the 4-point SMPG profile).
- Follow the predefined meals and document the consumption in the diary.
- Attend the clinical unit at approximately 15:00 hours on Day 4 of Visits 6 and 11.

8.1.6.2 Day 4 of V6 and V11

On Day 4 of the steady state periods, the subject should attend the clinical unit at approximately 15:00 hours. The subject will stay at the clinical unit for approximately 48 hours. Standardised meals will be given during the clinical unit admission and the carbohydrate intake should be recorded.

The following will be carried out:

- Check of withdrawal criteria
- Check of dosing day exclusion criteria (steady state period exclusion criteria)
- Check of concomitant medication
- From the diary, or from the subjects' medical records as applicable, the following information must be transcribed to the CRF:
  - Insulin treatment (name, dose, date and time)
  - Hypoglycaemic episodes
  - AEs
  - Changes in concomitant medication
  - SMPG values
- Alcohol breath test
- Urine pregnancy test (in women)
- Drug accountability

CGM will start on this day and continue until 24 hours after the start of the exercise bout (see section 8.4.2). The diary will not be used during the clinical unit admission; relevant information will instead be captured directly in the subject's medical record.

8.1.6.3 Day 5 of V6 and V11 (exercise bout day)

On Day 5 of each steady state period subjects will receive a standardised breakfast and a standardised lunch (at approximately 12:00 hours) at the clinical unit. The subjects should self-administer the IMP at approximately 07:00 hours (± 15 min). IAsp given at lunch will be reduced by 50% compared with the usual IAsp dose during the steady state period. The subjects should not do any other exercise than the ergometer exercise bout during the clinical trial unit admission on day 5 and 6 of Visits 6 and 11.

Before the start of the exercise bout, the following will be carried out:

- Check of withdrawal criteria
- Check of dosing day exclusion criteria (steady state period exclusion criteria)
- Check of concomitant medication
- Check of AEs
- Check of hyperglycaemic episodes
- Physical examination
- Vital signs
- Body weight
- Drug accountability
- The following information should be collected in the source data and must be transcribed to the CRF:
    - Insulin treatment (name, dose, date and time)
    - Hypoglycaemic episodes
    - AEs
    - Changes in concomitant medication
    - SMPG values

A vein will be cannulated for sampling of blood to assess blood glucose, counter-regulatory hormone concentrations and beta-hydroxybutyrate. Blood sampling will be performed before, during and after the exercise bout according to Table 2-2. The pre-exercise blood glucose value at Visit 6 Day 5, must be within the range of ≥ 5.0 mmol/l and ≤ 10.0 mmol/L in order to be eligible for the exercise bout. The pre-exercise blood glucose value for the second exercise bout (Visit 11) must be within 2 mmol/L above or below the pre-exercise value at the first exercise bout.

During and after exercise interstitial glucose (IG) will also be monitored by CGM (glucose values will not be visible).

At approximately 15:00 hours (3 hours after the standardised lunch), the subjects will perform 30 minutes ergometer bicycling at 65% $VO_{2\ peak}$.

Hypoglycaemic episodes as well as carbohydrate administration (type, amount and time) will be recorded during exercise and for the following 24 hours. For rescue criteria for hypo- and hyperglycaemic episodes during and after exercise, refer to section 8.4.1.1.

The IAsp dose in the evening can be adjusted (if needed) at the discretion of the investigator after evaluating the pre-meal BG value.

8.1.6.4 Day 6 of V6 and V11

Last dosing of IMP will take place at approximately 07:00 hours. Standard meals will be provided and carbohydrate intake recorded until discharge from the clinical unit at approximately 15:30 hours. Blood sampling will be performed according to Table 2-2. CGM will be stopped 24 hours after the start of the post-exercise.

The following will be carried out:
- Check of withdrawal criteria
- Check of dosing day exclusion criteria (steady state period exclusion criteria)
- Check of concomitant medication
- Check of AEs
- Check of hypoglycaemic episodes
- Check of insulin treatment (name, dose, date and time)
- Drug accountability
- Carbohydrate administration, if needed according to section 8.4.1.1 (until 24 hours following the start of the exercise)

At Visit 6, subjects should be asked to attend the clinical unit at approximately 06:30 hours in the morning of Visit 7. During the wash-out period, the subjects will resume their normal insulin treatment. Information on insulin treatment during the wash-out period should be given and NPH insulin provided if needed.

Subjects should also be reminded to comply with the requirements given in section 8.1.3 regarding exercise, diet, medication and insulin treatment prior to Visit 7.

8.1.7 Follow-up visit (Visit 12)

The subject will attend a follow-up visit, 7–21 days after Visit 11, Day 6.

The following will be assessed and recorded in the CRF:
- Body weight
- Physical examination
- Vital signs

- ECG
- Laboratory examination of blood/urine samples for safety assessment (haematology, biochemistry and urinalysis)
- Laboratory examination for pregnancy (in women)
- Check of AEs
- Check of Hypoglycaemic episodes
- Concomitant medication

In addition, the end of trial form must be completed.

8.2 Concomitant illness and medical history

A **concomitant illness** is any illness that is present at the start of the trial (i.e. at the screening visit). All concomitant illnesses should be reported.

Medical history: is an account of medical events that the subject has experienced in the past. Only relevant medical history should be reported.

The information collected for concomitant illness and relevant medical history should include diagnosis, date of onset, date of resolution or continuation.

Any change to a concomitant illness should be recorded during the trial, including eventually end date. A clinically significant worsening of a concomitant illness must be reported as an AE.

8.3 Concomitant medication

A **concomitant medication** is any medication, other than the IMPs and NIMPs, which is taken during the trial, including the screening, run-in and follow-up periods.

Details of any concomitant medication, including current insulin treatment, must be recorded at trial entry (i.e. at the first visit). Any changes in concomitant medication must be recorded at each visit as they occur.

The information collected for each concomitant medication includes (at a minimum) trade name or generic name, indication, start date and stop date or continuation.

If a change is due to an AE, then this must be recorded and reported according to section 11. If the change influences the subject’s eligibility to continue in the trial, the monitor must be informed.

8.4 Assessments for pharmacodynamics

A forearm vein will be cannulated with an 18-gauge polytetrafluoroethylene (PTFE) catheter for sampling of blood to assess blood glucose, counter-regulatory hormone and beta-hydroxybutyrate concentrations. The catheter will be kept open by use of a mandrin/stylet.

8.4.1 Blood glucose measurement

Before, during and after the exercise bout, blood glucose levels will be measured using a laboratory method (Super GL Glucose Analyzer; Dr Müller Gerätebau GmbH, Freital, Germany). Blood sampling will be performed according to Table 2-2. Allowed time windows are: Pre-exercise sample (-10 min) ± 5 min, pre-exercise sample (0 min) - 1 min, 0h to 30 min ± 1 min, 40 min to 3h ± 2 min, 4h30min to 22h30min: before standardised meals - 5 min, 90 min after the start of standardised meals ± 5 min, nominal time 7h and 13h post start of exercise ± 10 min.

8.4.1.1 Rescue criteria for hypo- and hyperglycaemic episodes during and after exercise

The following rescue criteria apply during and after the exercise bout (for 24 hours after the start of the exercise):

- If BG < 2.8 mmol/L or PG < 3.1 mmol/L a hypoglycaemic episode will be reported and oral carbohydrates will be provided and recorded (type, amount and time).
- If BG ≥ 16.0 mmol/L or PG ≥ 19.2 mmol/L bolus s.c. or i.v. IAsp will be administered.

If a blood glucose value corresponds to the above rescue criteria the blood glucose measurement will be repeated. If the repeated glucose measurement confirms that blood glucose is < 2.8 mmol/L or ≥ 16.0 mmol/L, the subject must be treated to alleviate the hypo- and hyperglycaemia symptoms according to the rescue criteria described above.

See also Section 8.5.5 for details on how to report hypoglycaemic episodes during the trial.

In case of unscheduled IAsp administration due to hyperglycaemia from pre-exercise to 24 hours post-exercise, the experiment should continue (decision on which data to include in the analysis will be taken subsequently).

8.4.1.2 Carbohydrate administration

If BG is < 2.8 mmol/L the subject will be given 10 g of carbohydrate (1 carbohydrate unit) e.g. 100 mL apple juice. A new BG value should be measured approximately 10 min after the carbohydrate administration. If the next BG value is still < 2.8 mmol/L the carbohydrate administration and BG measurement procedure will be repeated. Type, time and amount of carbohydrate intake will be recorded in the CRF. If the subject is unable to take oral carbohydrates, the subject will be treated according to best available medical practise, for example with i.v. glucose infusion or glucagon injection.

8.4.2 Continuous glucose monitoring (CGM)

CGM should be started after the subject has attended the clinical unit on Day 4 on Visits 6 and 11. It will continue until 24 hours after the start of the exercise bout.

A glucose sensor will be inserted under the skin, measuring the concentration of glucose in the surrounding interstitial fluid. The measurements are recorded by a small monitoring device worn by the subject. The Dexcom SEVEN Plus system will be used for CGM. Please refer to CGM Users Guide for how to use the CGM device and how to handle data. Calibration of the CGM device should follow manufacturer's guidelines. Venous blood should be used for calibration of the CGM devices.

IG values will not be visible (blinded) in order to not affect the behaviour of the subjects.

The IG values generated should not be used for recording of hypoglycaemic episodes. However, during the calibration of the CGM, any blood/plasma glucose values measured that qualify as a hypoglycaemic episode should be recorded.

In order to ensure that the CGM device is measuring correctly, the abdominal insulin injections must be in a distance of at least 8 cm from where the CGM sensor has been inserted.

8.4.3 Exercise bout

On Day 5 of each steady state period subjects will perform 30-minutes ergometer bicycling at 65% $VO_{2\ peak}$ at 15:00 hours, which is 3 hours after a standardised lunch. IAsp given at lunch will be reduced by 50% compared with the usual IAsp dose given during the steady state period.

Indirect calorimetry will be performed during the exercise bout. The subjects will wear a breathing mask and gas exchange will be measured breath-by-breath. Respiratory exchange ratio (RER) and substrate oxidation rates will be measured in order to assess carbohydrate and lipid oxidation during the exercise bout (see section 16.4.3). $\%VO_{2peak}$ will be calculated at three times two minutes to confirm that the subjects perform the exercise bout close to their 65% VO_{2peak} (see section 16.4.3).

The subjects should not do any other exercise than the ergometer exercise bout during the clinical trial unit admission on Day 5 and 6 of Visits 6 and 11.

Any hypoglycaemic episodes as well as carbohydrate administration (type, amount and time) will be recorded during and after the exercise bout (for 24 hours after the start of the exercise bout (0 min)).

8.4.4 Counter-regulatory hormones

Analysis of adrenaline, noradrenaline, cortisol, glucagon and growth hormone will be performed at pre-specified time points according to Table 2-2 by standard procedures at the local laboratory.

Allowed time windows are: Pre-exercise sample (0 min): - 1 min, 30 min: ± 1 min, 1 h to 3 h: ± 2 min.

8.4.5 Beta-hydroxybutyrate

Analysis of beta-hydroxybutyrate will be performed at pre-specified time points according to Table 2-2 by standard procedures at the local laboratory. Allowed time windows are: Pre-exercise sample (0 min): - 1 min, 30 min: ± 1 min, 1 h to 3h: ± 2 min.

8.5 Assessments for safety

8.5.1 Physical examination

An examination of the following body systems will be performed at pre-specified time points according to Table 2-1.

- Head, ears, eyes, nose, throat, neck
- Respiratory system
- Cardiovascular system
- Gastro-intestinal system incl. mouth
- Musculoskeletal system
- Central and peripheral nervous system
- Skin

At the screening visit, any abnormality will be recorded and described in the CRF including the investigator's assessment of clinical significance ('abnormal, not clinically significant' or 'abnormal, clinically significant'). Clinically significant findings at the screening visit should be recorded as concomitant illness.

At subsequent visits, any clinically significant deterioration of a pre-existing condition as well as any new clinically significant findings will be recorded as AEs.

8.5.2 Vital signs

Vital signs will be measured and recorded in the CRF at pre specified time points according to Table 2-1.

- Diastolic and systolic blood pressure (mmHg) measured after at least 5 minutes rest in a supine position.
- Pulse (beats per min) measured after at least 5 minutes rest in a supine position.
- Body temperature, ear (°C).

8.5.3 Electrocardiogram (ECG)

A standard 12-lead ECG with a rhythm strip will be performed at the screening and follow-up visits.

At the screening visit, a 1-minute rhythm strip will be recorded. At the follow-up visit, a 10 second rhythm strip (or at least 3 usable ECG intervals) will be recorded.

Any abnormality will be recorded and described in the CRF including the investigator's assessment of clinical significance ('abnormal, not clinically significant' or 'abnormal, clinically significant'). At the screening visit, clinically significant findings should be recorded as concomitant illness. Any clinically significant deterioration of a pre-existing condition as well as any new clinically significant findings in ECG will be recorded as AEs.

8.5.4 Self measured plasma glucose (SMPG)

Subjects will be instructed to perform self-measurement of PG during the two treatment periods (except during and 24 hours after the exercise bout).

The PG level should be measured and recorded in the diary and/or in the subject's medical record at the following time points:

- before breakfast
- before lunch
- before main evening meal
- at bedtime

During Day 1–4 of the steady state period, the PG level should also be measured 3 hours after lunch.

Subjects will be supplied with a glucose meter and instructions on how to use the device, including regular calibration according to the manufacturer's instructions. Written instructions will also be provided. The supplied glucose meters use test strips calibrated to PG values.

Subjects will be provided with a diary and instructed on how to record the SMPG values in the diary. The record of each SMPG value should include date, time and PG value. Occasional review by the investigator of the subject's PG values stored in the glucose meter's memory is advised to assure adequacy of the diary records. During the in-house periods SMPG measurements will be captured by the site staff in the subject's medical record. In case of discrepancies between the diaries/subject's medical record and the stored glucose meter values, only values from the diaries/ subject's medical record will be transcribed to the CRF.

8.5.5 Hypoglycaemic episodes

Plasma (or blood) glucose should always be measured and recorded when a hypoglycaemic episode is suspected.

All plasma glucose values:
- equal or below 3.9 mmol/L (70 mg/dL)

or
- higher than 3.9 mmol/L (70 mg/dL) when they occur in conjunction with hypoglycaemic symptoms, should be recorded by the subjects

These must be transcribed into the CRF (hypoglycaemic episode form) throughout the trial from Visit 1 to Visit 12.

The record should include the following information:
- The plasma/blood glucose level before treating the episode (if available)
- Date of hypoglycaemic episode
- Time of hypoglycaemic episode
- Whether the episode was symptomatic
- Whether the subject was able to treat him/herself
- Time of last insulin administration prior to episode
- Type of last insulin prior to episode
- Time of last main meal prior to episode
- Whether the episode occurred in relation to exercise

The answer to the question: “Was subject able to treat him/herself?” should be answered “No” if oral carbohydrates, glucagon or IV glucose had to be administered to the subject by another person because of severe central nervous system (CNS) dysfunction associated with the hypoglycaemic episode. Oral carbohydrates should not be given if the subject is unconscious.

A hypoglycaemic episode form must be filled in for each hypoglycaemic episode. If the hypoglycaemic episode fulfils the criteria for an SAE and/or a medical event of special interest (MESI) then an AE form and a safety information form must also be filled in.

8.5.6 ADA classification of hypoglycaemia

Severe hypoglycaemia: An episode requiring assistance of another person to actively administer carbohydrate, glucagon, or other resuscitative actions.

Asymptomatic hypoglycaemia: An episode not accompanied by typical symptoms of hypoglycaemia, but with a measured plasma glucose concentration ≤ 3.9 mmol/L (70 mg/dL).

Documented symptomatic hypoglycaemia: An episode during which typical symptoms of hypoglycaemia are accompanied by a measured plasma glucose concentration ≤ 3.9 mmol/L (70 mg/dL).

Relative hypoglycaemia: An episode during which the person with diabetes reports any of the typical symptoms of hypoglycaemia, and interprets those as indicative of hypoglycaemia, but with a measured plasma glucose concentration > 3.9 mmol/L (70 mg/dL).

Probable symptomatic hypoglycaemia: An episode during which symptoms of hypoglycaemia are not accompanied by a plasma glucose determination (but that was presumably caused by a plasma glucose concentration ≤ 3.9 mmol/L [70 mg/dL]).

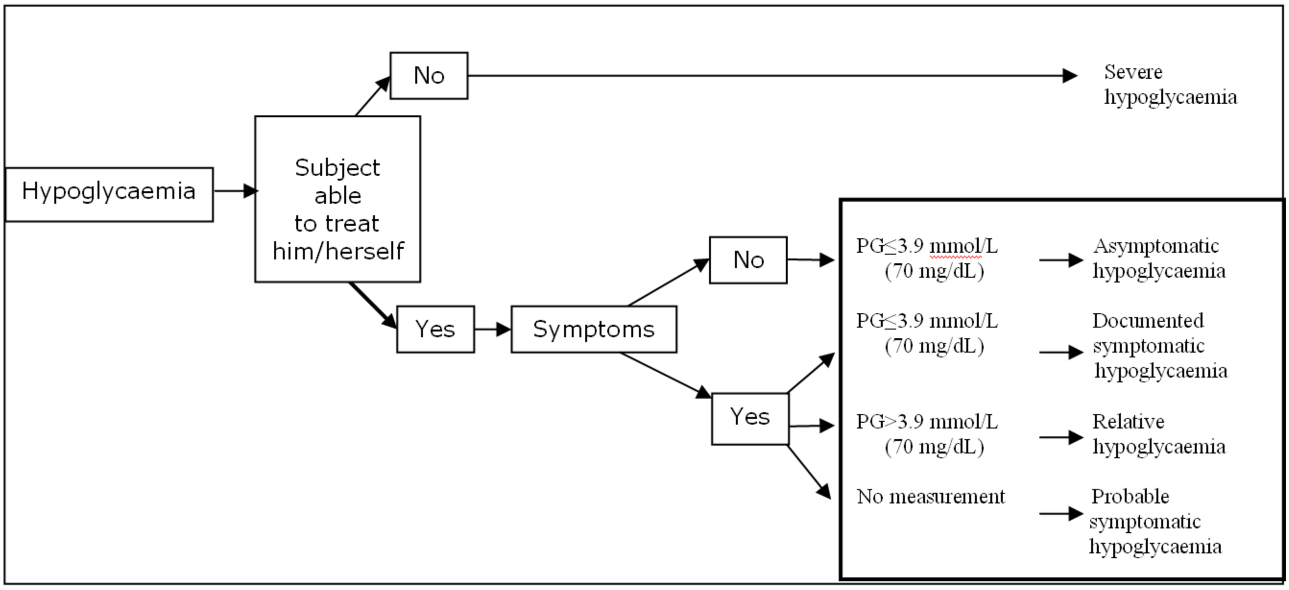

Figure 8-1 ADA classification of hypoglycaemia

8.5.7 Additional definitions of hypoglycaemia

A hypoglycaemic episode will be defined as treatment emergent if the onset of the episode occurs after the first administration of IMP, and no later than 7 days after the last day on trial product.

Hypoglycaemic episodes will be defined as nocturnal if the time of onset is between 00:01 and 05.59 inclusive.

In normal physiology, symptoms of hypoglycaemia occur below a blood glucose level of approximately 2.8 mmol/L (50 mg/dL) or plasma glucose level 3.1 mmol/L (56 mg/dL). Therefore, Novo Nordisk has used this cut-off point to define minor hypoglycaemia.

A minor hypoglycaemic episode is defined as either:

- An episode with symptoms consistent with hypoglycaemia with confirmation by blood glucose < 2.8 mmol/L (50 mg/dL) or plasma glucose < 3.1 mmol/L (56 mg/dL), and which is handled by the subject him/herself,

or

- Any asymptomatic blood glucose value < 2.8 mmol/L (50 mg/dL) or plasma glucose value < 3.1 mmol/L (56 mg/dL).

8.5.8 Adverse events

AEs will be recorded in accordance with the procedures described in Section 11.2. Any clinically significant worsening since baseline of a previous finding must be reported as an AE.

During each contact with the trial site staff (site visits and telephone contacts) the subject must be asked about AEs and technical complaints (TCs). This must be documented in the subject's medical record.

8.5.8.1 Adverse events describing injection site reactions

In case AEs describing injection site reactions are recorded, additional information should be collected by assessing the injection site reaction within the following categories:

- Spontaneous pain
- Pain on palpation
- Itching
- Redness
- Oedema
- Induration/infiltration
- Other

For each of these categories, a score of 0 (none), 1 (mild), 2 (moderate) or 3 (severe) will be reported. Time for start and end (or 'c' for continuing) of the reaction will also be recorded. Additional assessments will be performed until resolution, as judged necessary by the investigator. To ensure all local injection site assessments are performed at the injection site, the site of injection will be marked with a pen prior to each injection.

8.5.9 Laboratory assessments

Please, refer to Table 2-1 for exact timing of laboratory screening and safety assessments.

Measurement of HbA_{1c}, C-peptide, haematology, biochemistry, coagulation, hepatitis, HIV, pregnancy tests (blood), beta-hydroxybutyrate, counter-regulatory hormones and urinalysis will be performed by standard procedures at a local laboratory (see Attachment I).

The sampling, storage and shipment procedures are to be carried out according to the laboratory manual and instructions prepared by the laboratory.

Values outside the reference range will be marked by the laboratory on the laboratory prints. The investigator must evaluate all results outside the reference range (‘clinically significant’ or ‘not clinically significant’) and sign and date each page. The signed and dated version will be filed with the investigator’s trial documentation (with the subject’s medical record). If a result is considered clinically significant and it fulfils the criteria for a clinical laboratory AE (CLAE), it should be reported in accordance with Section 11.2. Clinically significant laboratory findings from the screening visit should be recorded as concomitant illness. The results of all tests performed by the laboratory will be transferred electronically to the data management unit within Novo Nordisk or external contract research organisation (CRO) performing data management (see Section 14).

Laboratory equipment in local laboratories may provide standard analyses not requested in the protocol but produced automatically in connection with the requested analyses. Such data will not be transferred to the CRF or the trial database, but must be reported to the investigator. The investigator must review all laboratory results for concomitant illnesses and AEs and report these according to this protocol.

8.5.9.1 HbA_{1c} and fasting C-peptide

Blood will be sampled for analysis of HbA_{1c} at the screening visit (to evaluate trial participation eligibility) and at Visit 7 (to capture any metabolic change after the first treatment period of the trial is completed). Blood will be sampled for analysis of fasting C-peptide at Visit 2.

8.5.9.2 Haematology

- Erythrocytes
- Haematocrit
- Haemoglobin
- Leucocytes
- Mean corpuscular haemoglobin (MCH)
- Mean corpuscular haemoglobin concentration (MCHC)
- Mean corpuscular volume (MCV)
- Thrombocytes

8.5.9.3 Biochemistry

- Aspartate aminotransferase (ASAT)
- Alanine aminotransferase (ALAT)
- Albumin
- Alkaline phosphatase (ALP)

- Bilirubins, total
- Chloride
- Calcium
- Creatinine
- Gamma glutamyltransferase (GGT)
- Phosphate
- Potassium
- Sodium
- Urea
- Uric acid

8.5.9.4 Coagulation

- Activated partial thromboplastin time (APTT)
- International normalised ratio (INR)

8.5.9.5 Urinalysis

The following analyses will be performed from a sample of mid-stream urine:

- Erythrocytes
- Glucose
- Ketones
- Leucocytes
- pH
- Protein

8.5.9.6 Hepatitis B and C and HIV

Serum screen on Hepatitis B antigen, Hepatitis C virus antibody, HIV-1 and HIV-2 antibodies and HIV-1 antigen (HIV 1/2 combi test) will be measured.

8.5.9.7 Pregnancy test

For women, a blood pregnancy test will be performed at the screening visit and at the follow-up visit.

At the other visits indicated in Table 2-1, a pregnancy test will be performed at the trial site using a urine stick (positive/negative outcome).

8.5.10 Alcohol breath test and screen for drugs

An alcohol breath test will be performed using an alcohol meter ($^{0}/_{00}$) by standard procedures at the trial site.

A urine test for the presence of amphetamine/ecstasy, benzodiazepines, cannabis, cocaine, methamphetamine, and opiates will be performed at the trial site from at least 5 mL fresh mid-stream urine using a stick.

8.6 Other assessments

8.6.1 Diagnosis of diabetes

- Date of diagnosis of diabetes

8.6.2 Demography

- Age
- Sex
- Ethnicity
- Race

8.6.3 Body measurements

- Height (m), without shoes
- Body weight (kg), only wearing underwear
- BMI in kg/m^2 calculated by the investigator based on height and body weight (body weight/height^2)

8.6.4 IPAQ

At the screening visit, the subject will be asked to fill out a short version of International Physical Activity Questionnaire (IPAQ) modified to cover physical activity during the last three months. The level of physical activity in MET-minutes will then be calculated according to Guidelines for Data Processing and Analysis of the IPAQ[8]. The level of physical activity will be transcribed into the CRF.

8.6.5 Indirect calorimetry

Indirect calorimetry will be performed at screening (refer to section 8.6.6) and during the exercise bouts at Visit 6 and 11 (refer to section 8.4.3).

8.6.6 VO_{2peak} test

At screening, each subject will perform a peak oxygen uptake (VO_{2peak}) test to assess endurance exercise capacity and trial participation eligibility. The assessment will be done by a cycle ergometer exercise. The aerobic physical fitness level of subjects will be measured with spiroergometry, which is a clinical exercise tolerance test with simultaneous respiratory gas measurements.

The cycle ergometer exercise bout will follow a step incremental protocol. The test will begin with a 3 minutes rest while sitting on the cycle ergometer. Then, the subjects will begin with 5 minutes baseline warm-up cycling (with little resistance), after which the step incremental exercise protocol will be initiated (30 Watt/3 min for women and 40 Watt/3 min for men). The subjects will continue exercising until volitional fatigue. Fatigue is defined as the inability to maintain a pedaling cadence at the minimum of 60 rpm (complete rotations per minute).

Ventilation and alveolar gas exchange are measured breath-by-breath throughout the test protocol. The subjects will be breathing through a facemask, which is connected to a low-resistance turbine and laboratory gas analyzer (Quark, Cosmed; Italy) for measurement of inspiratory and expiratory volumes, flow rate and for concentrations of O_2 and CO_2. The turbine will be calibrated before each test by using a syringe of known volume (3.00 L). The O_2 and CO_2 sensors will be calibrated in the morning of a test using a reference precision analyzed gas mixture.

VO_{2peak} will be determined as the highest measured VO_2 value over a 30 sec period (a 30 sec moving average will be used).

BG measurements will be performed prior and after the test and carbohydrates will be available for the subjects if needed. The subjects will be under constant medical care during the test.

8.7 Volume of blood to be drawn during the trial

The planned total volume of blood to be drawn from each subject during the trial will amount to a maximum of 160 mL.

8.8 Subject compliance

The investigator will assess the compliance of the subject at each visit based on a review of glycaemic control, adherence to the visit schedule and completion of subject diaries including SMPG data. Regarding trial products, the investigator will assess the unused amount of trial products against the prescribed amount and, in case of discrepancies, question the subject.

At each visit the investigator will remind the subjects to comply with protocol procedures.

9 Trial supplies

Trial supplies comprise trial products and auxiliary supplies. Details are included in the trial materials manual (TMM).

9.1 Trial products

Trial products comprise IMPs. Auxiliary supplies comprise supplies other than trial products. Trial products must not be dispensed to any person not included in the trial.

The following IMPs will be used:
- Insulin degludec (100 U/mL) in 3 mL pre-filled investigational pens (PDS290).
- Insulin glargine (Lantus®), 100 U/mL in 3 mL SoloStar®.

The IMPs will be provided by Novo Nordisk A/S, Denmark.

9.2 Non-investigational medicinal products

The following NIMPs will be used:
- Insulin aspart (NovoRapid®), 100 U/mL in 3 mL FlexPen®.
- Neutral Protamine Hagedorn (NPH) insulin (Prothaphane®), 100 IU/ml in 3 ml FlexPen®.

The NIMPs will be provided by the investigator.

9.3 Labelling

Labelling of the IMPs will be in accordance with Annex 13[14], local law and trial requirements. Novo Nordisk will repack the outer package and re-label commercially available Lantus® as described in the TMM.

Enough trial products will be packed for the planned number of subjects. A buffer volume of trial product will be provided in case of replacement of withdrawn subjects or damage to trial product.

9.4 Storage, accountability and destruction

Trial products (both not-in use and when in use) should not be exposed to excessive heat or direct sunlight. Trial products must not be used if it does not appear clear and colourless. Please refer to the TMM for further information about trial product handling at the trial site.

Storage conditions for the unopened trial products:

- Store in a refrigerator 2°C–8°C
- Do not freeze
- Protect from light

- Do not use after the expiration date

Storage conditions for the open (in-use) trial products:

IDeg 100 U/mL (PDS290):
- Do not refrigerate
- Do not store above 30°C
- Use within 8 weeks of being open

IGlar (Lantus®), (SoloStar®):
- Do not refrigerate
- Keep at room temperature (below 30°C)
- Use within 28 days of being open

Storage and handling of the NIMPs should be in accordance with locally approved labels.

The investigator must ensure the availability of proper storage conditions, and record and evaluate the temperature. During storage, the temperature must be logged and evaluated at least once every working day. Minimum requirement is a calibrated minimum/maximum thermometer. The temperature recorder should be either electronic with a minimum interval of logging of 1 hour or manual with a min-max measuring device, which can be either mechanic or electronic. A temperature log must be kept to document storage within the right temperature interval. The temperature reading must be transferred to the temperature log every working day. Storage facilities should be checked frequently (at least once every working day). The investigator must inform Novo Nordisk immediately if any trial product has been stored outside defined conditions (e.g. outside temperature range). The investigator must take appropriate action to avoid recurrence of the detected deviation. Trial products that have been stored improperly must not be dispensed to any subject before it has been re-evaluated and approved for further use by Novo Nordisk.

A randomisation list will be used when trial products are allocated to the subjects. The subjects are instructed to return all used, partly used and unused trial product at the end of each treatment period (Visits 6 and 11). Returned trial products (used/partly used or unused including empty packaging material) must be stored separately from non-allocated trial products.

Trial product accountability must be performed. The accountability will include the number of allocated, returned and lost pen devices. Responsibility for trial product accountability at the trial site rests with the investigator. The tasks can be delegated to a study nurse or a pharmacist.

The accountability for NIMPs (IAsp and NPH insulin) also rests with the investigator and constitutes of a registration of received and used product, and total daily dose for each subject (at

every visit to the site). Dose information must be source data verifiable and should be collected in the CRF.

For NIMPs, trial ID, batch no, expiry date and storage conditions will be recorded in a separate drug receipt form.

Upon completion of the clinical trial report (CTR), the investigator is responsible for destruction of IMPs according to local procedures after drug accountability is finalised at the trial site. The monitor will provide instruction of the destruction procedure for the trial product. The trial product should only be destroyed upon instruction from the monitor. The destruction must be documented in the investigator file. Destruction of NIMPs may be done at the end of the trial (LSLV) after drug accountability is finalised at the trial site.

9.5 Auxiliary supply

The following auxiliaries will be provided by the investigator:

- Blood glucose meters (incl. lancets, test strips and control solution for calibration)
- NovoFine® needles (for PDS290 and FlexPen®)
- Needles for SoloStar®
- Equipment for CGM
- Equipment needed to perform the VO_{2peak} test
- Equipment needed to perform the exercise bout
- All equipment needed for performing urinalysis and for sampling of blood for the laboratory screening and safety assessments
- Oral carbohydrates (during and after exercise)
- IAsp for i.v. infusion (during and after exercise)
- Equipment needed for performing the pregnancy urine dip stick test and the urine drug screen
- Materials necessary for sampling of blood for analysis of C-peptide as described in the relevant laboratory manual
- ID cards
- Subject diaries

The following auxiliaries will be provided by the local laboratory:

- Equipment needed for performing the pregnancy urine dip stick test and the urine drug screen as agreed between the investigator and the local laboratory and described in the laboratory manual
- Laboratory manual

10 Randomisation procedure

This is a randomised trial. The randomisation will be provided by Clinical Supplies Coordination, Novo Nordisk A/S, Denmark. Treatment sequence allocation will take place at the trial site. The randomisation list will be delivered directly to an unblinded pharmacist at the site in order not to reveal the treatment sequence to the investigator before randomisation.

Subjects will receive IDeg and IGlar in randomised sequence (1:1). When a subject is randomised in the trial, he/she must always be assigned the lowest available randomisation number. A replacement subject must be allocated to the same treatment sequence as the subject he/she replaces, using the corresponding replacement number.

Randomisation and replacement numbers will be:

- Randomisation numbers: 101-140
- Replacement numbers: 201-240

11 Adverse events, technical complaints and pregnancies

11.1 Definitions

An **adverse event** (AE) is any untoward medical occurrence in a subject administered a product, and which does not necessarily have a causal relationship with this treatment.

An AE can therefore be any unfavourable and unintended sign (including an abnormal laboratory finding), symptom or disease temporally associated with the use of a product, whether or not considered related to the product.

This includes events from the first trial related activity after the subject has signed the informed consent and until the follow-up period.

AEs include:

- A clinically significant worsening of a concomitant illness.
- A clinical laboratory adverse event (CLAE): A clinical laboratory abnormality which is clinically significant, i.e. an abnormality that suggests a disease and/or organ toxicity and is of a severity that requires active management. Active management includes active treatment or further investigations, for example change of medicine dose or more frequent follow-up due to the abnormality.

The following should **not** be reported as AEs:

- Pre-existing conditions, including those found as a result of screening procedures (pre-existing conditions should be reported as medical history or concomitant illness).
- Pre-planned procedures unless the condition for which the procedure was planned has worsened from the first trial related activity after the subject has signed the informed consent.
- Non-serious hypoglycaemia are AEs, but are reported on hypoglycaemic forms instead of on AE forms.

An AE is either a serious AE (SAE) or a non-serious AE.

An **SAE** is an experience that at any dose results in any of the following:

- Death
- A life-threatening[a] experience
- In-patient hospitalisation[b] or prolongation of existing hospitalisation
- A persistent or significant disability or incapacity[c]
- A congenital anomaly or birth defect
- Important medical events that may not result in death, be life threatening[a] or require hospitalisation[b] may be considered an SAE - when based on appropriate medical judgement -

they may jeopardise the subject and may require medical or surgical intervention to prevent one of the outcomes listed in this definition.[d]

a The term "life threatening" in the definition of SAE refers to an event in which the subject was at risk of death at the time of the event. It does not refer to an event which hypothetically might have caused death if it was more severe.

b The term "hospitalisation" is used when a subject:

- Is admitted to a hospital or in-patient, irrespective of the duration of physical stay, or
- Stays at the hospital for treatment or observation for more than 24 hours

Medical judgement must always be exercised, and when in doubt, the hospital contact should be regarded as a hospitalisation. Hospitalisations for administrative, trial related and social purposes do not constitute AEs and should therefore not be reported as AEs or SAEs. Hospital admissions for surgical procedures, planned before trial inclusion, are not considered AEs.

c A substantial disruption of a subject's ability to conduct normal life functions (e.g. following the event or clinical investigation the subject has significant, persistent or permanent change, impairment, damage or disruption in his/her body function or structure, physical activity and/or quality of life).

d For example intensive treatment in an emergency room or at home of allergic bronchospasm, blood dyscrasiasis or convulsions that do not result in hospitalisation, or development of drug dependency or drug abuse.

A **non-serious AE** is any AE which does not fulfil the definition of an SAE.

A **medical event of special interest** (MESI) is an event which, in the evaluation of safety, has a special focus. A MESI should be reported according to the same reporting requirements and timelines as for SAEs (see section 11.2) irrespective of whether the MESI fulfils any SAE criterion.

The following are defined as MESIs in this trial:

1. Medication errors concerning trial products (including NIMPs):

- Administration of wrong drug
- Wrong route of administration, such as intramuscular instead of subcutaneous
- Administration of a high dose with the intention to cause harm (eg suicide attempt)
- Administration of an accidental overdose

  "that is, a dose which may lead to significant health consequences, as judged by the investigator, irrespective of whether any SAE criterion is fulfilled"

2. Suspected transmission of an infectious agent via a trial product

Severity assessment definitions:
- **Mild** – no or transient symptoms; no interference with the subject's daily activities.
- **Moderate** – marked symptoms; moderate interference with the subject's daily activities.
- **Severe** – considerable interference with the subject's daily activities; unacceptable.

The following terms and definitions are used when assessing the relationship between each AE and the relevant trial products (including NIMPs):
- **Probable** - Good reason and sufficient documentation to assume a causal relationship
- **Possible** - A causal relationship is conceivable and cannot be dismissed
- **Unlikely** - The event is most likely related to aetiology other than the trial product

The following terms and definitions are used in assessing the final outcome of an AE:
- **Recovered** - The subject has fully recovered, or by medical or surgical treatment the condition has returned to the level observed at the first trial-related activity after the subject signed the informed consent.
- **Recovering** - This term is only applicable if the subject has completed the trial or has died from another AE. The condition is improving and the subject is expected to recover from the event.
- **Recovered** with sequelae - The subject has recovered from the condition, but with lasting effect due to a disease, injury, treatment or procedure. If a sequela meets an SAE criterion, the AE must be reported as an SAE.
- **Not recovered** - The condition of the subject has not improved and the symptoms are unchanged, or the outcome is not known at the time of reporting.
- **Fatal** - This term is only applicable if the subject died from a condition related to the reported AE. Outcomes of other reported AEs in a subject before he/she died should be assessed as "recovered", "recovering", "recovered with sequelae" or "not recovered". An AE with fatal outcome must be reported as an SAE.
- **Unknown** - This term is only applicable if the subject is lost to follow-up.

A **technical complaint** is any communication that alleges defects on trial supplies. The technical complaint may be associated with an AE, but does not concern the AE itself.

Examples of technical complaints:
- The physical or chemical appearance of trial products (e.g. discoloration, particles or contamination)
- The packaging material (e.g. leakage, cracks, rubber membrane issues or errors in labelling text)
- Problems related to devices (e.g. to the injection mechanism, dose setting mechanism, glucose measurement, push button or interface between the pen and the needle)

11.2 Reporting of adverse events

All events meeting the definition of an AE must be collected and reported. This includes events from the first trial-related activity after the subject has signed the informed consent until the end of the post-treatment follow-up period. The events must be recorded in the applicable forms in a timely manner.

During each contact with the trial site staff (site visits and telephone contacts), the subject must be asked about AEs and technical complaints. This must be documented in the subject's medical record. The subject will be asked: "Have you experienced any problems since the last contact?"

All AEs, either observed by the investigator or reported by the subject, must be reported by the investigator and evaluated. Novo Nordisk assessment of expectedness is performed according to the following reference documents:

- Insulin degludec: The current edition of the investigator´s brochure[3], or any updates thereto
- Insulin glargine (Lantus®): Current version of the EU Summary of Product Characteristics[15]

The investigator should report the diagnosis, if available. If no diagnosis is available, the investigator should record each sign and symptom as an individual AE.

All AEs must be recorded by the investigator on the AE form. A separate AE form should be used for each diagnosis or sign and symptom. For each SAE a safety information form should be completed in addition to the standard AE form. If several symptoms or diagnoses occur as part of the same clinical picture, one safety information form may be used to describe all the SAEs.

MESIs, regardless of seriousness, must be reported using both the AE form and the safety information form.

The active post-treatment follow-up period will continue until the follow-up visit (Visit 12). During this period, new AEs should be recorded to ensure that information on withdrawal effects is available.

The investigator must ensure that the worst case severity and seriousness of an event is kept throughout the trial.

The investigator must complete and forward the following forms to Novo Nordisk either electronically (e.g. in PDF format) or by fax:

- AE form within 24 hours of obtaining knowledge of the SAE or MESI
- Safety information form **within 5 calendar** days of obtaining knowledge of the SAE or MESI

Contact details (fax, telephone, e-mail and address) are provided in Attachment I to the protocol.

Novo Nordisk will notify the investigator of trial product-related suspected unexpected serious adverse reactions (SUSARs) in accordance with local requirements and ICH Good Clinical Practice (GCP)[16]. In addition, the investigator will be informed of any trial-related SAEs that may warrant a change to any trial procedure.

Novo Nordisk must always inform the regulatory authorities in accordance with local requirements and GCP.

11.3 Follow-up of adverse events

All SAEs and MESIs must be followed up until the outcome of the event is “recovered”, “recovered with sequelae” or “fatal”, and until all queries have been resolved. Cases of chronic conditions, cancer or AEs ongoing at time of death (where death is due to another AE) may be closed with the outcome “recovering” or “not recovered”, when the subject has completed the follow-up period.

The follow-up information should only include new (corrections or new or additional) information and should be reported **within 24 hours** of obtaining knowledge of the information. This is also the case for previously non-serious AEs which subsequently become SAEs.

Non-serious AEs must be followed until the outcome of the event is “recovering”, “recovered” or “recovered with sequelae” or until the end of the follow-up period stated in the protocol, whichever comes first, and until all queries related to these AEs have been resolved. Cases of chronic conditions or cancer or AEs ongoing at time of death (where death is due to another AE) may be closed with the outcome “recovering” or “not recovered”.

Queries or follow-up requests from Novo Nordisk should be responded to within 13 (or less) calendar days.

The investigator must forward follow-up information on SAEs and MESIs to Novo Nordisk within 24 hours of obtaining the follow-up information by using a new AE form and/or a safety information form marked as follow-up.

The investigator must forward follow-up information on non-serious AEs as corrections to the original AE form, or by using a new AE form marked as follow-up.

11.4 Technical complaints and technical complaint samples

11.4.1 Reporting of technical complaints

All technical complaints on any of the following products (IMPs) which occur from the time of first usage of trial supplies until the time of the last usage of trial supplies must be collected and reported to Novo Nordisk.

The investigator must assess whether the technical complaint is related to any AE(s), SAE(s) and/or MESI(s).

Technical complaints must be reported on a separate technical complaint form and must be completed for each IMP listed on the technical complaint form. If the technical complaint involves more than one batch number, a technical complaint form for each batch number must be completed.

The investigator must complete and forward the technical complaint form by fax, e-mail or courier to Novo Nordisk, within the same timelines as for reporting AEs, SAEs and MESIs as follows:

- Technical complaint assessed as related to an SAE and/or MESI within 24 hours of the trial site obtaining knowledge of the complaint
- All other technical complaints within 5 calendar days

11.4.2 Collection, storage and shipment of technical complaint samples

The investigator must collect the technical complaint sample and notify the monitor within 5 calendar days. The monitor must initiate the shipment to Novo Nordisk and ensure the sample is sent in accordance with local regulations and as soon as possible to Novo Nordisk complaint centre. A copy or a print of the technical complaint form should be sent with the sample.

The investigator should ensure that the technical complaint sample contains the batch number and, if available, the dispensing unit number (DUN).

If the technical complaint sample is unobtainable, the investigator must specify on the technical complaint form why it is unobtainable.

Storage of the technical complaint sample must be done in accordance with the conditions prescribed for the product (see section 9 and the TMM). The shipment of the technical complaint sample should be done in accordance with the same conditions as for storage.

11.5 Pregnancies

When an abnormality is reported in the foetus or newborn infant, information is needed from the male partner. Informed consent must be obtained prior to this.

Female subjects must be instructed to notify the investigator immediately if they become pregnant during the trial. The investigator must report any pregnancy in subjects who received Novo Nordisk provided trial product.

The investigator must report all information on pregnancies, including AEs in the subject, the foetus, and newborn infant on the trial related pregnancy forms. The pregnancy forms must be forwarded to Novo Nordisk preferably electronically in PDF format or by fax.

The investigator must follow the pregnancy until the pregnancy outcome and the newborn infant(s) until the age of one month. The investigator must collect information on the pregnancy and pregnancy complications as well as the pregnancy outcome including the health of the newborn infant(s) on the pregnancy forms.

The following must be collected:

- Initial information **within 14 calendar days of the investigator's first knowledge of the pregnancy**
- Information on the outcome of her pregnancy - including the health status of the newborn infant at the age of one month **within 14 calendar days of the investigator's knowledge of the pregnancy outcome**
- All non-serious **AEs** in connection with the pregnancy and pregnancy outcome must be reported on the pregnancy forms within 14 calendar days of the investigator's knowledge. It must be clear in the description if the event occurs in the subject, the foetus or the newborn infant.
- All **SAEs** in connection with the pregnancy and pregnancy outcome must be reported on the pregnancy forms following the **same timelines as required for other SAEs** (see section 11.2). It must be clear in the description if the event occurs in the subject foetus or the newborn infant.
  - The SAEs that must be reported include abnormal outcome - such as congenital anomalies, foetal death and termination of pregnancy (spontaneous or elective abortion), including any anomalies of the foetus observed at gross examination or during autopsy - as well as other pregnancy complications fulfilling the criteria of an SAE.

11.6 Precautions and/or overdose

Normal precautions taken for human trials, including the provision of emergency equipment, will be taken during this trial. Qualified and well-trained physicians and medical staff will instruct the subjects.

The most common side effect of all available insulin products is hypoglycaemic episodes (too low blood sugar). This type of side effect can be mild with just a few symptoms: cold sweat, hunger, headache, nausea, light-headedness, palpitations (increased pulse/heart beat), dizziness, tremor (slight shaking), weakness and difficulties in concentrating. A hypoglycaemic episode can potentially in very rare cases be more severe with unconsciousness and even death.

Mild to moderate symptoms of hypoglycaemia, or blood glucose less than 3.5 mmol/L (63 mg/dL), can be treated by ingestion of carbohydrate (for example apple juice). Severe hypoglycaemia resulting in loss of consciousness should be treated at the investigator's discretion according to best available medical practice.

Insulin or the preservatives in the insulin products may give rise to allergic reactions. Local allergic reactions (such as redness, swelling and itching at the injection site) may occur but usually disappear after a few weeks.

11.7 Committees related to safety

11.7.1 Novo Nordisk safety committee

Novo Nordisk will constitute an internal safety committee to perform ongoing safety surveillance.

12 Case report forms

Paper CRFs will be provided by Novo Nordisk A/S.

Print legibly, using a ballpoint pen. Ensure that all questions are answered, and that no empty data blocks exist. Ensure that no information is recorded outside the data blocks.

If a test/assessment has not been done and will not be available, indicate this by writing “ND” (not done) in the appropriate answer field in the CRF. If the question is irrelevant (e.g. is not applicable) indicate this by writing “NA” (not applicable) in the appropriate answer field. Further guidance can be obtained from the instructions in the CRF.

The investigator must ensure that all information derived from source documentation is consistent with the source information. By signing the affirmation statement, the investigator confirms that the information in the CRF including related forms are complete and correct.

12.1 Corrections to case report forms

Corrections to the data in CRFs may only be made by drawing a straight line through the incorrect data and then writing the correct entry next to the data that were crossed out. Each correction must be initialled, dated and explained (if necessary) by the investigator or the investigator’s authorised staff. If corrections are made by the investigator’s authorised staff after the date of the investigator’s signature on the affirmation statement, the affirmation statement must be signed and dated again by the investigator.

Corrections necessary after the CRFs have been removed from the investigator’s site must be documented on a data clarification form (DCF) or a monitor-initiated discrepancy form (MIDF). If the affirmation statement for the subject has not yet been signed, any corrections must be approved by the investigator or her/his authorised staff. If the affirmation statement for the subject has already been signed, the investigator must approve any correction.

12.2 Case report form flow

The investigator must ensure that data are recorded in the CRF as soon as possible after the visit.

CRFs are produced on NCR (No Carbon Required) paper with one original and one copy. Following monitoring, the top page (original page) of the CRF is transferred by the monitor to the data management unit within Novo Nordisk or external CRO performing data management (see Section 14). The copy is to be archived with the investigator’s trial documentation. The investigator should sign the affirmation statement. All corrections in the CRF and resolution of queries must be dated before the date of the affirmation statement. If corrections and/or resolution of queries are dated after signed affirmation statement, the statement should be signed and dated again by the investigator.

The investigator will receive the safety laboratory printouts (the laboratory report) directly from the laboratory. The investigator must review, evaluate, sign and date the laboratory report immediately upon receipt. The signed and dated version will be filed together with the investigator's trial documentation (with the subject's medical record).

The investigator must review, evaluate, sign and date the ECGs. The signed and dated versions of the ECGs will be filed together with the investigator's trial documentation (with the subject's medical record).

13 Monitoring procedures

During the course of the trial, the monitor will visit the trial site to ensure that the CRFs are completed correctly and the protocol adhered to, to perform source data verification, monitor drug accountability and collect completed CRF pages. Monitoring visit intervals will not exceed 4 weeks.

The monitor must be given direct access to source documents (original documents, data and records). Direct access includes permission to examine, analyse, verify and reproduce any record(s) and report(s) that are important to the evaluation of the trial. If the electronic medical record does not have a visible audit trail, the investigator must provide the monitor with signed and dated printouts. In addition the relevant trial site staff should be available for discussions at monitoring visits and between monitoring visits (e.g. by telephone).

The monitor will collect CRF pages and other trial related forms containing data from screening failures. The screening failure form and AE forms, if any, are monitored, source data verified and entered into the trial database. All other data from screening failure subjects are not required to be entered into the trial database.

All data must be verifiable in source documentation other than the CRF.

For withdrawals, all available CRF data should be monitored, source data verified and entered into the database. Withdrawn subjects should attend a follow-up visit and Visit 12 procedures should be performed. The CRF pages covering the follow-up visit should be completed together with the end of trial form.

Evaluated and signed laboratory printouts as well as evaluated and signed ECGs and copies of the ECGs must be inserted in the subject's medical record at the site.

There must be a source document agreement at each site. There should only be one source defined at any time for any data element. Diaries and IPAQ originals must not be removed from the site.

A detailed diagram and description of the transmission of electronic data should be provided. The source data and their respective capture methods should be clearly defined.

The monitor will ensure that the CRFs are completed and that paper CRFs are collected.

Monitors must review the medical records and other source data (diaries and IPAQ) to ensure consistency and/or identify omissions compared to the CRF. If discrepancies are found, the investigator must be questioned about these.

14 Data management

Data management is always the responsibility of Novo Nordisk. Data management may be delegated under an agreement of transfer of responsibilities to another data management unit within Novo Nordisk or an external CRO.

Laboratory data from the local laboratory will be transferred electronically. In cases where laboratory data are transferred via non-secure electronic networks, data will be encrypted during transfer.

The subject and any biological material obtained from the subject will be identified by subject ID and trial identification number. Appropriate measures such as encryption or leaving out certain identifiers will be enforced to protect the identity of subjects in all presentations and publications as required by local, regional and national requirements.

The following will be transferred to Novo Nordisk:

From the local laboratory:

- HbA_{1c} and serum C-peptide
- Haematology
- Biochemistry
- Coagulation
- Urinalysis
- Hepatitis and HIV tests
- Blood pregnancy test
- Counter-regulatory hormone concentrations
- Beta-hydroxybutyrate concentrations

From the investigator:

- Blood glucose concentrations (from Super GL Glucose Analyzer)
- IG values (from CGM)

15 Computerised systems

Novo Nordisk will capture and process clinical data using computerised systems that are described in Novo Nordisk Standard Operating Procedures (SOPs) and IT architecture documentation. The use and control of these systems are documented.

Investigators working on the trial may use their own electronic systems to capture source data. Novo Nordisk will collect information on the practical use of these systems within the conduct of this clinical trial.

16 Statistical considerations

Novo Nordisk A/S will be responsible for the statistical analyses and if necessary, a statistical analysis plan (SAP) may be written in addition to the protocol, including a more technical and detailed elaboration of the statistical analyses. The SAP will be finalised before database lock (DBL).

All pharmacodynamic (PD) endpoints will be summarised by treatment using descriptive statistics. Moreover, complete listings of individual values for all endpoints and parameters will be provided.

Individual and mean curves for the glucose profiles will be plotted by treatment over the sampling period.

Presentation of results from a statistical analysis will include the estimated mean for each treatment. Estimated mean treatment differences (or ratios) will be presented together with two-sided 95% confidence intervals for all endpoints analysed statistically.

Subject disposition will be tabulated including the numbers of screened subjects, screening failures, subjects exposed to IMP, withdrawals including reason, subjects completing the trial, subjects in the full analysis set (FAS) and subjects in the Safety Analysis Set (see Section 16.2 for the definition of these analysis sets).

Subjects withdrawn from the trial will be listed including the primary reason for withdrawal.

Data collected before exposure will be summarised using descriptive statistics.

16.1 Sample size calculation

Using data from NN304-1724 (insulin detemir exercise trial), the within subject standard deviation (StD) of the baseline adjusted $BG_{pre\text{-}exe}$ - $BG_{minimum,exe}$ is estimated to 0.88, 0.93 and 0.96 respectively for the three treatments in NN304-1724 (IGlar, NPH and insulin detemir). The standard deviation for the treatment difference of the baseline adjusted $BG_{pre\text{-}exe}$ - $BG_{minimum,exe}$ between IDeg and IGlar is thereby expected to be in the range of 1.25 and 1.35.

The results of the sample size calculations can be seen in table Table 16-1 for different standard deviations and mean treatment difference that can be detected. A significance level of 5% and a power of 80% are used.

Table 16-1 Sample size calculation

Mean treatment difference (mmol/L)	StD=1.25	StD=1.35
0.5	52	60
0.65	32	36
0.8	22	26

Assuming a standard deviation of 1.35, a treatment difference of 0.65 mmol/L (judged as clinically relevant) can be detected with 80% power if 36 subjects complete the trial. In order to account for dropouts, a total of 40 subjects should be randomised.

16.2 Definition of analysis sets

The following analysis sets are defined in accordance with the ICH-E9 guidance:[17]

- Full Analysis Set (FAS): includes all randomised subjects. In exceptional cases subjects from the FAS may be eliminated. In such cases the elimination will be justified and documented. Subjects will contribute to the evaluation "as treated".
- Safety Analysis Set: includes all subjects receiving at least one dose of one of the IMPs. Subjects in the safety set will contribute to the evaluation "as treated".

Analyses of PD endpoints will be based on the FAS. The analysis of the safety endpoints will be based on the Safety Analysis Set.

Before data are released for statistical analysis, a review of all data will take place. Furthermore, extreme values and outliers will be identified by the Trial Statistician during programming and data review according to ICH-E9[17]. In addition, protocol deviations are reviewed in order to identify deviations, which may potentially affect the results.

Obviously erroneous data points may be excluded from the analyses. The decision to exclude data points from the statistical analysis is the joint responsibility of the Clinical Pharmacology Scientist and the Trial Statistician.

The subjects or observations to be excluded and the reason for their exclusion will be documented and signed by the Clinical Pharmacology Scientist and the Trial Statistician, prior to DBL. The documentation will be stored together with the remaining trial documentation. The subjects and observations excluded from analysis sets, and the reason for this, will also be described in the CTR.

The impact of protocol deviations and outliers may be investigated further in sensitivity analyses if deemed relevant.

16.3 Primary endpoint

The following endpoint will be derived from the individual blood glucose concentration profile:

- $BG_{pre\text{-}exe}$ - $BG_{minimum,exe}$, difference between blood glucose concentration before exercise and the minimum blood glucose concentration observed during exercise (from 0 to 30 minutes).

16.3.1 Analysis of primary endpoint

The primary endpoint will be analysed in a linear mixed model with $BG_{pre\text{-}exe}$ as a covariate, treatment and period as fixed factors and subject as a random effect. In order to account for potential heteroscedasticity, the within-subject variation will depend on the treatment.

In this model, the treatment difference (IDeg vs. IGlar) will be estimated together with the 95% confidence interval.

16.4 Secondary endpoints

16.4.1 Secondary pharmacodynamic endpoints

The secondary PD endpoints for the exercise period presented in Table 16-2 will be derived from the individual blood glucose profile, hypoglycaemic episodes and carbohydrate administrations during exercise.

Table 16-2 Overview of pharmacodynamic endpoints for exercise period

Symbol	Definition	Details
$BG_{mean,exe}$	Mean blood glucose concentration during exercise (from 0 to 30 minutes)	The mean of all valid blood glucose measurements between 0 to 30 minutes.
$BG_{pre\text{-}exe}$ - $BG_{30min,exe}$	Difference between blood glucose concentration before exercise and after 30 minutes of exercise	None
$Hypo_{exe}$	Number of minor and severe hypoglycaemic episodes during exercise (from 0 to 30 minutes)	Minor hypoglycaemic episodes: episodes of PG < 3.1 mmol/L (56mg/dL)

Symbol	Definition	Details
CHO_{exe}	Number of carbohydrate administrations given during exercise (from 0 to 30 minutes) in response to hypoglycaemia.	None

The secondary PD endpoints for the period following exercise presented in Table 16-3 will be derived from the individual blood glucose profile, hypoglycaemic episodes and carbohydrate administrations after exercise.

Table 16-3 Overview of pharmacodynamic endpoints for period following exercise

Symbol	Definition	Details
$BG_{mean,30–180min,post-exe}$	Mean blood glucose concentration between 30 and 180 minutes, i.e. post-exercise	The mean of all valid blood glucose measurements between 30 to 180 minutes.
$BG_{minimum,30–180min,post-exe}$	Minimum blood glucose concentration between 30 and 180 minutes, i.e. post-exercise	The minimum of all valid blood glucose measurements between 30 to 180 minutes.
$tBG_{minimum,30–180min,post-exe}$	Time from end of exercise until minimum blood glucose concentration between 30 and 180 minutes, i.e. post-exercise	Time to the minimum of all valid blood glucose measurements. If not uniquely determined, the lowest value is chosen.
$Hypo_{24h,post-exe}$	Number of minor and severe hypoglycaemic episodes post-exercise (for 24 hours after start of exercise)	Minor hypoglycaemic episodes: episodes of PG < 3.1 mmol/L (56mg/dL).
Nocturnal $hypo_{24h,post-exe}$	Number of nocturnal minor and severe hypoglycaemic episodes post-exercise (for 24 hours after start of exercise)	Minor hypoglycaemic episodes: episodes of PG < 3.1 mmol/L (56mg/dL).<br>Nocturnal: period between 00:01 and 05:59 a.m. (both included).

Symbol	Definition	Details
$CHO_{24h,post\text{-}exe}$	Number of carbohydrate administrations given post-exercise (for 24 hours after the start of exercise) in response to hypoglycaemia	None

16.4.2 Analysis of secondary pharmacodynamic endpoints

$BG_{mean,exe}$, $BG_{mean,30–180min,post\text{-}exe}$ and $BG_{minimum,30–180min,post\text{-}exe}$ will be analysed in the same way as the primary endpoint. $BG_{pre\text{-}exe}$ - $BG_{30min,exe}$, and $tBG_{minimum,30–180min,post\text{-}exe}$ will be summarised descriptively.

$Hypo_{exe}$, $Hypo_{24h,post\text{-}exe}$, and nocturnal $hypo_{24h,post\text{-}exe}$ will be analysed in a negative binomial regression model with a log-link function if a sufficient number of hypoglycaemic episodes / carbohydrate administrations are obtained. The model will include treatment and period as fixed factors and subject as a random effect.

CHO_{exe}, and $CHO_{24h,post\text{-}exe}$ will be summarised descriptively.

16.4.3 Other pharmacodynamic parameters and measurements

The other PD parameters presented in Table 16-4 will be derived from the individual blood glucose profiles.

Table 16-4 Overview of other pharmacodynamic parameters

Symbol	Definition	Details
$BG_{minimum,exe}$	Minimum blood glucose concentration between 0 and 30 minutes	The minimum of all valid blood glucose measurements between 0 to 30 minutes
$BG_{30min,exe}$	Blood glucose concentration after 30 minutes of exercise	None

Regarding the following PD measurements, no pre-planned derived parameters will be calculated.

- CGM, IG profile based on CGM
- Counter-regulatory hormone concentrations (glucagon, adrenaline, noradrenaline, cortisol and growth hormone)
- Beta-hydroxybutyrate concentration
- HbA_{1c}

Regarding exercise bout, the following measurements and parameter will be recorded and derived:

- VO_2 (at 4–6 min, 9–11 min, 14–16 min, 19–21 min, 24–26 min and 28–30 min)
- RER (at 4–6 min, 9–11 min, 14–16 min, 19–21 min, 24–26 min and 28–30 min)
- $\%VO_{2peak}$, calculated as 100*VO_2 during exercise (at 9–11 min, 19–21 min and 28–30 min) divided by VO_{2peak}

16.4.4 Analysis of other pharmacodynamic parameters and measurements

$BG_{minimum,exe}$, and $BG_{30min,exe}$ will be summarised descriptively. Blood glucose at each time point and its change from baseline will be summarised descriptively. The change of blood glucose will be graphically presented.

Beta-hydroxybutyrate concentration will be summarised descriptively.

HbA_{1c} and its change from baseline will be summarised descriptively.

IG profiles based on CGM will be graphically presented.

Counter-regulatory hormone concentrations (glucagon, adrenalin, cortisol, noradrenalin and growth hormone) will be summarised descriptively.

VO_2, RER and $\%VO_{2peak}$ will be summarised descriptively.

16.4.5 Safety parameters

The following safety parameters will be assessed:

- Incidence of AEs
- Incidence of treatment emergent hypoglycaemic episodes
- Change in vital signs, ECG evaluations and laboratory safety variables

Adverse events

All AEs will be coded using the latest version of Medical Dictionary for Regulatory Activities (MedDRA). Individual AEs will be presented in listings including relation to individual IMPs/NIMPs and severity. Treatment emergent AEs (the onset of the AE is after first IMP administration and no later than 7 days after the last IMP administration) will be presented in a summary table including number of AEs, number of subjects with at least 1 AE and percentage of exposed subjects with at least 1 AE. A summary table will also be made by system organ class and treatment.

All local injection site reactions scored as mild, moderate or severe will be listed by subject number, insulin preparation, time-point, severity and duration. Moreover, local tolerability at the injection site will be summarised using descriptive statistics as appropriate.

Hypoglycaemic episodes

The number of treatment emergent hypoglycaemic episodes (the onset of the hypoglycaemic episode is after first IMP administration and no later than 7 days after the last IMP administration) and the percentage of subjects with at least one episode will be summarised. Separate summaries will be based on the severity (see Section 8.5.6) and the timing (day-time or nocturnal), if appropriate. Confirmed hypoglycaemic episodes are defined as minor (see Section 8.5.7) and severe hypoglycaemic episodes.

Laboratory safety variables

Laboratory values (biochemistry, coagulation, haematology and urinalysis) will be flagged if outside the reference range.

A listing of abnormal values will be presented. Laboratory parameters will be summarised at screening and at the follow-up visit.

Vital signs

Vital signs (diastolic and systolic blood pressure, pulse and body temperature) will be summarised using descriptive statistics.

ECG

The investigator's evaluations of ECGs will be summarised and abnormal individual evaluations will be listed together with the investigator's comments. Any clinical significant deterioration of a pre-existing condition after the screening visit, as well as any new clinically significant findings will be recorded as AEs.

17 Ethics

The trial will be conducted in compliance with ICH GCP[16] and applicable regulatory requirements, and in accordance with the Declaration of Helsinki[18].

The trial product may be associated with AEs (see Section 11) but relevant precautions have been implemented in the design and planned conduct of the trial in order to minimise the risks and inconveniences of participating in the trial. Furthermore, subjects are fully informed about possible AEs and inconveniences. The subjects will have the right to withdraw from the trial at any time, without giving a specific reason. The subjects can expect no benefits from participating except a thorough medical examination at the beginning and end of the trial and a close contact to a doctor in the treatment period.

At termination of the trial, the care of the subject will continue as previously. Novo Nordisk will not offer free drug at the end of the trial.

17.1 Informed consent

In seeking and documenting informed consent, the investigator must comply with applicable regulatory requirement(s) and adhere to ICH GCP[16] and the requirements in the Declaration of Helsinki[18].

Before any trial-related activity, the investigator must give the subject oral and written information about the trial in a form that the subject can read and understand.

The investigator must ensure the subject ample time to come to a decision whether or not to participate in the trial.

A voluntary, signed and personally dated informed consent form will be obtained from the subject before any trial-related activity.

The responsibility for seeking informed consent must remain with the investigator, but the task may be delegated by the investigator to a medically qualified person, in accordance with local requirements. The written informed consent must be signed and personally dated by the person who seeks the informed consent.

If information becomes available that may be relevant to the subject's willingness to continue participating in the trial, the investigator must inform the subject in a timely manner, and a revised written informed consent must be obtained.

17.2 Data handling

If the subject is withdrawn from the trial or lost to follow up, then the subject's data will be handled as follows:

- Data already collected will be retained by Novo Nordisk, entered into the database and used for the trial report
- Safety events will be reported to the Novo Nordisk and regulatory authorities according to local/national requirements

If data are used, it will always be in accordance with local law and Institutional Review Board (IRBs)/independent ethics committees (IECs).

17.3 Premature termination of the trial and/or trial site

Novo Nordisk, the investigator, the IRBs/IECs or a regulatory authority may decide to stop the trial, part of the trial or a trial site at any time, but agreement on procedures to be followed must be obtained.

If a trial is suspended or prematurely terminated, the investigator must inform the subjects promptly and ensure appropriate therapy and follow-up. The investigator and/or Novo Nordisk should also promptly inform the IRBs/IECs and provide a detailed written explanation. The relevant regulatory authorities should be informed.

If, after the termination of the trial, the risk/benefit analysis changes, the new evaluation should be provided to the IRBs/IECs in case it has an impact on the planned follow-up of subjects who have participated in the trial. If it does have an impact, the actions needed to inform and protect the subjects should be described.

18 Protocol compliance

Deviations from the protocol should be avoided.

If deviations do occur, the investigator must inform the monitor and the implications of the deviation must be reviewed and discussed.

Investigator must document and explain protocol deviations by stating the reason, date, and the action(s) taken.

Documentation on all protocol deviations must be kept in the investigator's trial file and Novo Nordisk trial master file.

19 Audits and inspections

Any aspect of the clinical trial may be subject to audits conducted by Novo Nordisk or inspections from domestic or foreign regulatory authorities or from IRBs/IECs. Audits and inspections may take place during and after the trial. The investigator and the site staff as well as Novo Nordisk staff have an obligation to cooperate and assist in such audits and inspections. This includes giving auditors and inspectors direct access to all source documents and other documents at the site relevant to the clinical trial. This includes permission to examine, analyse, verify and reproduce any record(s) and report(s) that are relevant to the evaluation of the trial.

20 Critical documents

Before a site is allowed to start screening subjects, the following documents must be available to Novo Nordisk:

- Regulatory approval and/or acknowledgement of notification as required
- Curricula vitae of investigator and sub-investigator(s) (current, dated and signed and/or supported by an official regulatory document. Must include documented GCP training or a certificate)
- Signed receipt of investigator's brochure
- Signed and dated agreement on the final protocol
- Signed and dated agreement on any substantial protocol amendment, if applicable
- Approval/favourable opinion from IRBs/IECs clearly identifying the documents reviewed as follows: protocol, any substantial protocol amendments, subject information/informed consent form, any other written information to be provided to the subject and subject recruitment materials
- List of IRB/IEC members and/or constitution
- Financial agreement(s)
- Source document agreement
- Local laboratory certification(s) and normal ranges
- Insurance statement, if applicable
- Signed and dated investigator agreement
- Financial disclosure form for all investigators

Novo Nordisk will analyse and report data.

From the local laboratory, the following documents must be available to Novo Nordisk:

- Laboratory reference ranges
- Laboratory certification/QA scheme/other documentation
- Laboratory methods

As documented in writing by protocol signature, each investigator agrees to comply fully with ICH standards of current Good Clinical Practice (GCP), applicable regulatory requirements and the declaration of Helsinki.

21 Responsibilities

All staff (Novo Nordisk, site, laboratory, CRO etc) will conduct the trial in compliance with ICH GCP[16], applicable regulatory requirements and the Declaration of Helsinki[18].

The investigator is accountable for the conduct of the trial at his/her site. If any tasks are delegated, the investigator should maintain a list of appropriately qualified persons to whom he/she has delegated specified significant trial-related duties. The investigator should ensure that there is adequate training for all staff participating in the conduct of the trial. It is the investigator's responsibility to supervise the conduct of the trial and to protect the rights, safety, and well-being of the subjects.

A qualified physician, who is an investigator or a sub-investigator for the trial, must be responsible for all trial-related medical decisions.

The investigator must ensure adequate supervision of the conduct of the trial at the trial site.

The investigator will follow instructions from Novo Nordisk when processing data.

The investigator is responsible for filing essential documents (i.e. those documents which individually and collectively permit evaluation of the conduct of a trial and the quality of the data produced) in the investigator's trial file. The documents should be kept in a secure locked facility, so no unauthorized persons can get access to the data. The subject ID list should be kept securely and separate from the personal data.

The investigator will take all necessary technical and organisational safety measures to prevent accidental or wrongful destruction, loss or deterioration of data. The investigator will prevent any unauthorised access to data or any other processing of data against applicable law.

The investigator must be able to provide the necessary information or otherwise demonstrate to Novo Nordisk that such technical and organisational safety measures have been taken.

During any period of unavailability, the investigator should delegate responsibility for medical care of subjects to a specific qualified physician who will be readily available to subjects during that time.

If the investigator is no longer able to fulfil the role of investigator (e.g. if he/she retires), a new investigator will be appointed in consultation with Novo Nordisk.

The investigator and site personel must have sufficient English skills according to their assigned task(s).

It is the responsibility of Novo Nordisk A/S to:

- Write the protocol.
- Review and approve the English version of the subject information, informed consent, subject diary and modified IPAQ.
- Prepare the Investigational Medicinal Product Dossier (IMPD) and assist in preparing the clinical trial application (CTA) documents.
- Ensure preparation of the CRF.
- Review and approve the CRF.
- Provide the IMPs and randomisation list.
- Prepare the SAP, if applicable.
- Perform data entry and data management.
- Approve the clean database.
- Perform statistical analysis of all data and prepare the CTR.

It is the responsibility of Novo Nordisk Pharma GmbH to:

- Review and approve the protocol.
- Review the subject information and informed consent in English and in local language. Approve the subject information in local language.
- Review the subject diary and modified IPAQ in English and in local language. Approve the subject diary and modified IPAQ in local language.
- Review the CRF.
- Collaborate with [redacted] on preparation of the CTA documents and review the package for CTA submission.
- Obtain 'Regulatory Green Light'.
- Perform monitoring and local trial management during the trial period.

It is the responsibility of [redacted] to:

- Review and approve the protocol.
- Write the subject information/informed consent form in English and in local language.
- Write the subject diary in English and in local language.
- Write a modified IPAQ in English and in local language.
- Provide a meal-plan (containing composition of standardised meals).
- Prepare and submit the CTA to the Ethics Committee and Health Authorities after sign off from Novo Nordisk.
- Provide the NIMPs
- Review the CRF.
- Provide auxiliaries as described in Section 9.5.
- Provide the data from the local laboratory and the PD data in oracle clinical (OC) loadable files.
- Review the CTR.

It is the responsibility of the local laboratory to:

- Provide a laboratory manual.
- Provide laboratory documentation as specified in Section 20 for the laboratory analyses.
- Provide auxiliaries as described in Section 9.5.
- Analyse the laboratory samples and provide the analysis results to [redacted].

22 Reports and publications

The information obtained during the conduct of this trial is considered confidential, and may be used by Novo Nordisk for regulatory purposes and for the general development of the trial product. All information supplied by Novo Nordisk in connection with this trial shall remain the sole property of Novo Nordisk and is to be considered confidential information. No confidential information shall be disclosed to others without prior written consent from Novo Nordisk. Such information shall not be used except in the performance of this trial. The information obtained during this trial may be made available to other physicians who are conducting other clinical trials with the trial product, if deemed necessary by Novo Nordisk.

Dr. [redacted] will be the principal investigator of the trial and will review and sign the CTR (i.e. [redacted] is the signatory investigator) on behalf of [redacted].

22.1 Communication of results

No permission to publish shall be granted to any clinical research organisation involved in the trial.

Novo Nordisk commits to communicating, and otherwise making available for public disclosure, results of trials regardless of outcome. Public disclosure includes publication of a paper in a scientific journal, abstract submission with a poster or oral presentation at a scientific meeting, or disclosure by other means.

Novo Nordisk reserves the right to defer the release of data until specified milestones are reached, for example when the clinical trial report is available. This includes the right not to release the results of interim analyses, because the release of such information may invalidate the results of the entire trial.

At the end of the trial, one or more public disclosures may be prepared collaboratively by the investigator(s) and Novo Nordisk. Novo Nordisk reserves the right to postpone publication and/or communication for up to 60 days to protect intellectual property.

The results of this trial will be subject to public disclosure on external web sites according to international regulations, as reflected in the Novo Nordisk Code of Conduct for Clinical Trial Disclosure.

In all cases the trial results will be reported in an objective, accurate, balanced and complete manner, with a discussion of the strengths and limitations. All authors will be given the relevant statistical tables, figures, and reports needed to support the planned publication. In the event of any disagreement on the content of any publication, both the investigators' and Novo Nordisk opinions will be fairly and sufficiently represented in the publication.

22.1.1 Authorship

Authorship of publications should be in accordance with the Uniform Requirements of the International Committee of Medical Journal Editors (sometimes referred to as the Vancouver Criteria[11]).

22.2 Investigator access to data and review of results

As owner of the trial database, Novo Nordisk has the discretion to determine who will have access to the database.

23 Retention of clinical trial documentation

Subject records must be kept for the maximum period permitted by the hospital, institution or private practice.

The investigator must agree to archive the documentation (this includes both electronic and paper-based records) pertaining to the trial in an archive after completion or discontinuation of the trial if not otherwise notified. The investigator should not destroy any documents without prior permission from Novo Nordisk. If the investigator cannot archive the documents at the trial site, Novo Nordisk can refer the investigator to an independent archive provider that has a system in place to allow only the investigator to access the files.

The investigator must be able to access his/her trial documents without involving Novo Nordisk in any way. Site-specific CRFs and other subject data (in an electronic readable format or as paper copies or prints) will be provided to the investigator before access is revoked to the systems and/or electronic devices supplied by Novo Nordisk. These data must be retained by the site. If the Novo Nordisk provided data (e.g. the CD-ROM) is not readable during the entire storage period, the investigator can request a new copy, as a copy of all data will be stored by Novo Nordisk.

Novo Nordisk will maintain Novo Nordisk documentation pertaining to the trial for as long as the product is on the market plus 20 years.

The files from the investigator site/institution must be retained for 15 years after the completion of the trial, or longer if required by national regulations. The deletion process must ensure confidentiality of data and must be done in accordance with local requirements.

24 Institutional Review Boards/Independent Ethics Committees and regulatory authorities

Written approval or favourable opinion must be obtained from IRB/IEC prior to commencement of the trial.

During the trial, the investigator or sponsor, as applicable, must promptly report the following to the IRB/IEC, in accordance with local requirements: updates to investigator's brochure, unexpected SAEs where a causal relationship cannot be ruled out, substantial protocol amendments, non-substantial protocol amendments according to local requirements, deviations to the protocol implemented to eliminate immediate hazards to the subjects, new information that may affect adversely the safety of the subjects or the conduct of the trial (including new risk/benefit analysis in case it will have an impact on the planned follow-up of the subjects), annually written summaries of the trial status, and other documents as required by the local IRB/IEC.

Substantial protocol amendments must not be implemented before approval or favourable opinion, unless necessary to eliminate immediate hazards to the subjects.

The investigator must maintain an accurate and complete record of all submissions made to the IRB/IEC. The records should be filed in the investigator's trial file and copies must be sent to Novo Nordisk.

Regulatory Authorities

Regulatory authorities will receive the CTA, substantial/non-substantial protocol amendments, reports on SAEs, and the CTR according to national requirements.

25 Indemnity statement

Novo Nordisk carries product liability for its products, and liability is assumed under the special laws, acts and/or guidelines for conducting clinical trials in any country, unless others have shown negligence.

Novo Nordisk assumes no liability in the event of negligence, or any other liability by the clinics or doctors conducting experiments, or by persons for whom the said clinic or doctors are responsible.

Novo Nordisk accepts liability in accordance with:

- The German drug law dated August 24, 1976 last amended by the 15th amendment of the drug law dated July 19, 2011.

26 References

1 The effect of intensive treatment of diabetes on the development and progression of long-term complications in insulin-dependent diabetes mellitus. The Diabetes Control and Complications Trial Research Group. N Engl J Med 1993; 329(14):977-986.

2 Intensive blood-glucose control with sulphonylureas or insulin compared with conventional treatment and risk of complications in patients with type 2 diabetes (UKPDS 33). UK Prospective Diabetes Study (UKPDS) Group. Lancet 1998; 352(9131):837-853.

3 Investigator's Brochure, insulin degludec (7th Edition). Novo Nordisk A/S. 1-Nov-2011.

4 O'Gorman DJ, Krook A. Exercise and the treatment of diabetes and obesity. Med Clin North Am 2011; 95(5):953-969.

5 Riddell MC, Perkins BA. Type 1 diabetes and vigorous exercise: applications of exercise physiology to patient management. Canadian Journal of Diabetes 2006; 30(1):63-71.

6 Yki-Jarvinen H, DeFronzo RA, Koivisto VA. Normalization of insulin sensitivity in type I diabetic subjects by physical training during insulin pump therapy. Diabetes Care 1984; 7(6):520-527.

7 Colberg SR, Sigal RJ, Fernhall B, Regensteiner JG, Blissmer BJ, Rubin RR et al. Exercise and type 2 diabetes: the American College of Sports Medicine and the American Diabetes Association: joint position statement. Diabetes Care 2010; 33(12):e147-e167.

8 Guidelines for Data Processing and Analysis of the International Physical Activity Questionnaire (IPAQ) - Short and Long Forms. 2005. Available from: www.ipaq.ki.se/scoring.pdf.

9 US Department of Health and Human Services. 2008 Physical Activity Guidelines for Americans [Internet]. 2012. Washington (DC), ODPHP Publication No. U0036. 2008 [cited 2010 Oct 10]. 61 p. Available from: http://www.health.gov/paguidelines/pdf/paguide.pdf.

10 The Criteria Committee of the New York Heart Association. Nomenclature and Criteria for Diagnosis of Diseases of the Heart and Great Vessels. 9th ed., 253-256. 1994. Boston, Mass: Little, Brown & Co.

11 De Angelius C, Drazen JM, Frizelle FA, Haug C, Hoey J, Horton R et al. Clinical trial registration: a statement from the International Committee of Medical Journal Editors. New Engl J Med 2004; 351(12):1250-1251.

12 Food and Drug Administration. Food and Drug Administration Amendments Act of 2007. 2007.

13 European Commission Regulation for EudraCT. 2010.

14 European Union. The rules governing medicinal products in the European Union, volume 4, Annex 13, Manufacture of Investigational Products - Brussels. 2003.

15 Lantus - Summary of Product Characteristics. 2010.

16 International Conference on Harmonisation. ICH Harmonised Tripartite Guideline: Guideline for Good Clinical Practice E6 (R1), Step 4. 10-6-1996.

17 International Conference on Harmonisation. ICH Harmonised Tripartite Guideline. Statistical principles for clinical trials E9. International Conference on Harmonisation E9 Expert Working Group. 5-2-1998.

18 World Medical Association. World Medical Association Declaration of Helsinki: Ethical principles for medical research involving human subjects - Last amended by the 59th WMA General Assembly, Seoul. 2008.
